# Supplementary material for: The Intriguing Effects of Substituents in the N-Phenethyl Moiety of Norhydromorphone: A Bifunctional Opioid from a Set of “Tail Wags Dog” Experiments
Source: Molecules. 2020 Jun 6;25(11):2640. doi: 10.3390/molecules25112640 (PMC7321161; doi:10.3390/molecules25112640)

molecules

Supplementary Materials

The Intriguing Effects of Substituents in the *N*-Phenethyl Moiety of Norhydromorphone: A Bifunctional Opioid from a Set of “Tail Wags Dog” Experiments

Meining Wang [1], Thomas C. Irvin [1], Christine A. Herdman [1], Ramsey D. Hanna[1], Sergio A. Hassan [2], Yong-Sok Lee [2], Sophia Kaska [3], Rachel Saylor Crowley [3], Thomas E. Prisinzano [3], Sarah L. Withey [4], Carol A. Paronis [4], Jack Bergman [4], Saadet Inan [5], Ellen B. Geller [5], Martin W. Adler [5], Theresa A. Kopajtic [6], Jonathan L. Katz [6], Aaron M. Chadderdon [7], John R. Traynor [7], Arthur E. Jacobson [1,*] and Kenner C. Rice [1,*]

[1] Department of Health and Human Services, Drug Design and Synthesis Section, Molecular Targets and Medications Discovery Branch, Intramural Research Program, National Institute on Drug Abuse and the National Institute on Alcohol Abuse and Alcoholism, National Institutes of Health, 9800 Medical Center Drive, Bethesda, MD 20892-3373, USA; wangmeining8911@163.com (M.W.); Tom.irvin8@gmail.com (T.C.I.); caherdman@gmail.com (C.A.H.); Ramsey.hanna1@gmail.com (R.D.H.)

[2] Department of Health and Human Services, Center for Molecular Modeling, Office of Intramural Research, Center for Information Technology, National Institutes of Health, Bethesda, MD 20892, USA; hassan@mail.nih.gov (S.A.H.); leeys@mail.nih.gov (Y.-S.L.)

[3] Department of Medicinal Chemistry, School of Pharmacy, University of Kansas, Lawrence, KS 66045-7582, USA; sophia.kaska@uky.edu (S.K.); crowleyrachels@gmail.com (R.S.C.); prisinzano@uky.edu (T.E.P.)

[4] McLean Hospital/Harvard Medical School, 115 Mill Street, Belmont MA 02478, USA; swithey@mclean.harvard.edu (S.L.W.); cparonis@mclean.harvard.edu (C.A.P.); jbergman@hms.harvard.edu (J.B.)

[5] Lewis Katz School of Medicine of Temple University, 3500 N. Broad St., Philadelphia, PA 19140, USA; sinan@temple.edu (S.I.); gellerellen@gmail.com (E.B.G.); baldeagl@temple.edu (M.W.A.)

[6] Department of Health and Human Services, Psychobiology Section, Molecular Neuropsychiatry Research Branch, Intramural Research Program, National Institute on Drug Abuse, National Institutes of Health, Baltimore, MD 21224, USA; theresa.kopajtic@gmail.com (T.A.K.); jkatzzz@gmail.com (J.L.K.)

[7] Department of Pharmacology and Edward F Domino Research Center, University of Michigan Medical School, Ann Arbor, MI 48109, USA; Aaron.Chadderdon@gmail.com (A.M.C.); traynor@umich.edu (J.R.T.)

* Correspondence: arthurj@nida.nih.gov (A.E.J.); kennerr@nida.nih.gov (K.C.R.); Tel.: 301-451-5028 (A.E.J.); Tel.: 301-451-4799 (K.C.R.)

Table of Contents: **Page #**

1) ^{1}H & ^{13}C NMR 4-20

2) Quantum chemical calculations and molecular dynamics simulations

Table S1 21

Fig. S1 22

Fig. S2 23

Table S2 25

Fig. S3 26

3) **Scheme S1**. Synthesis of Hydromorphone (**S5**), *N*-Norhydromorphone (**S10**) and *N*-Phenethylnorhydromorphone (**S11**) 27

^{1}H and ^{13}C NMR of **1a**

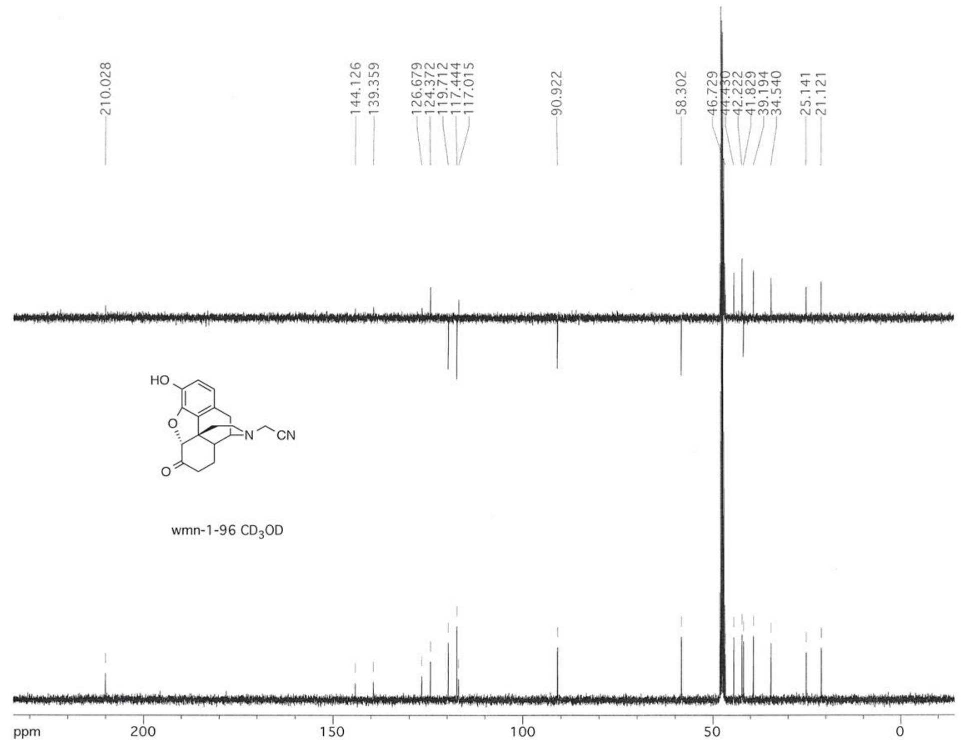

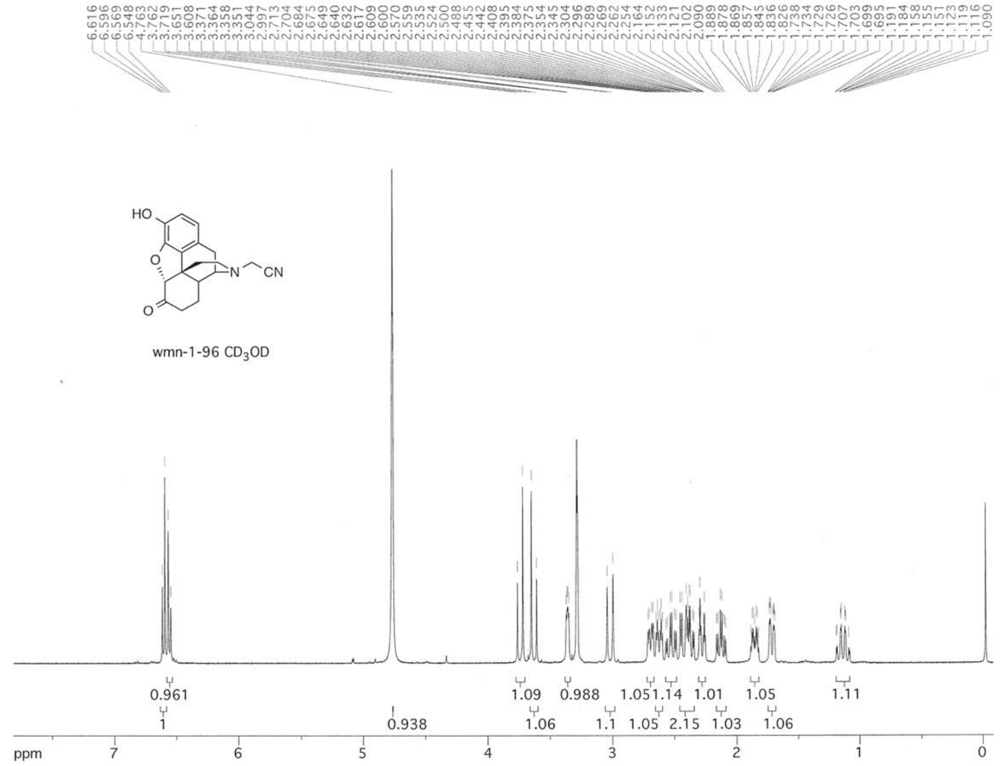

^{1}H and ^{13}C NMR of **1b**

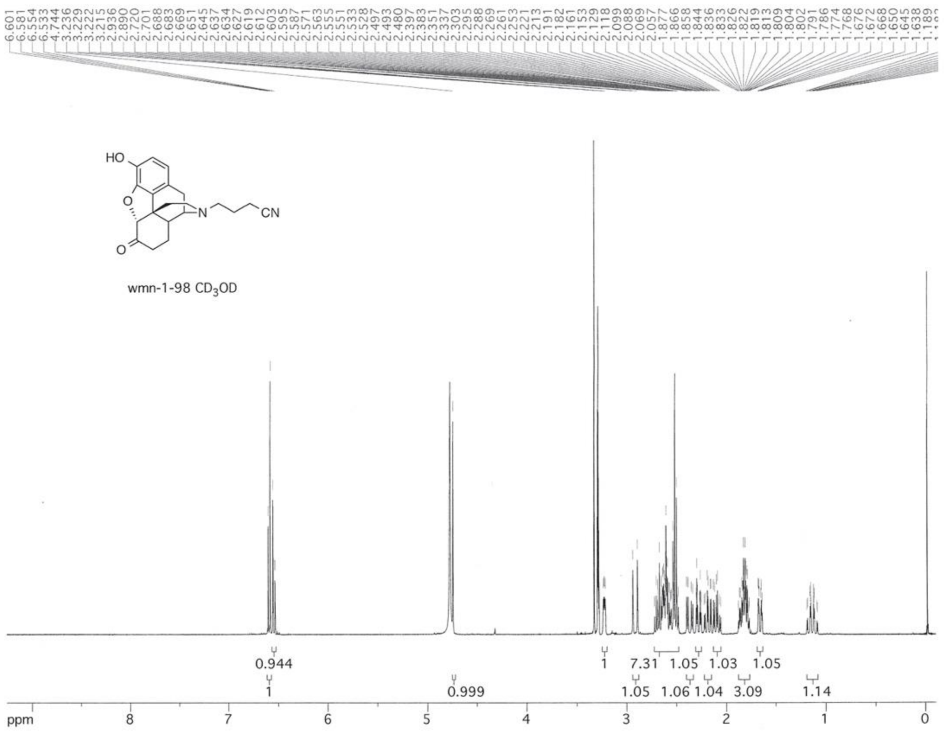

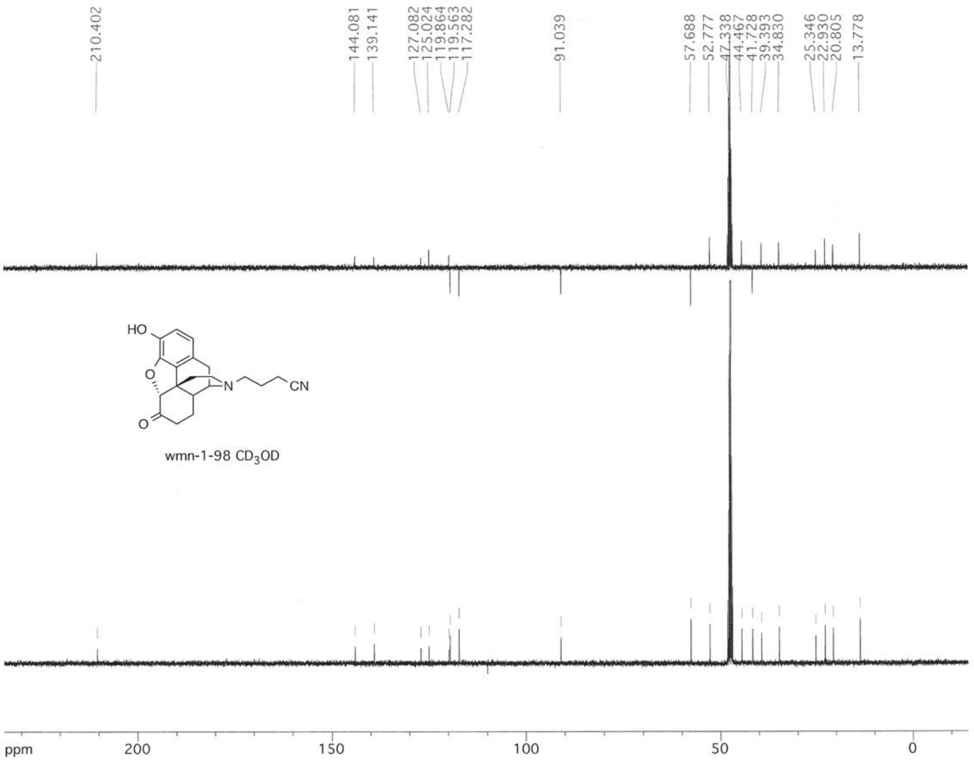

^{1}H and ^{13}C NMR of **1c**

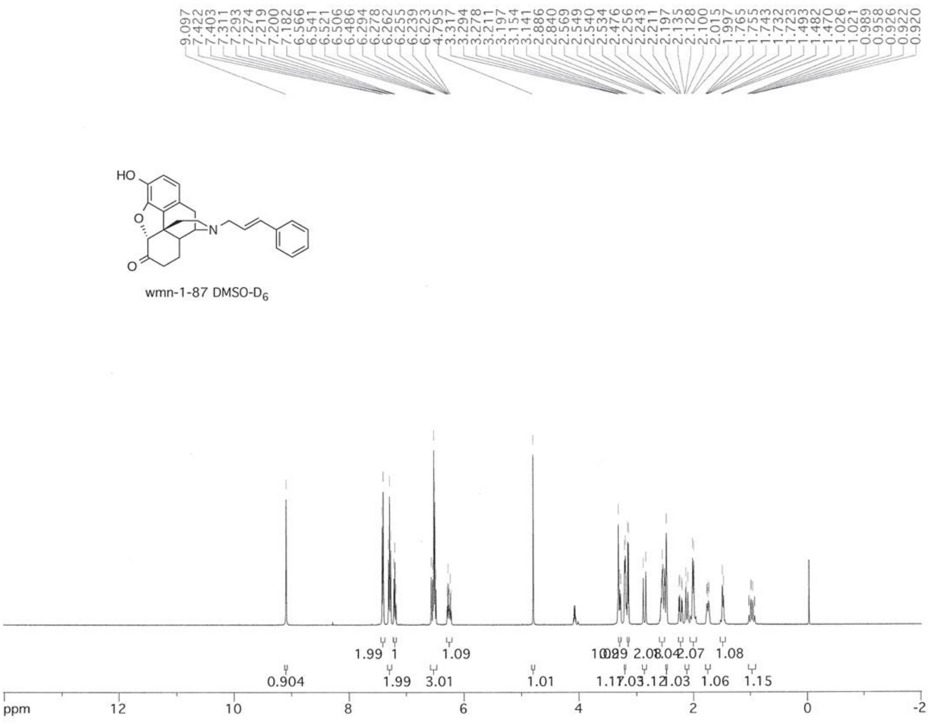

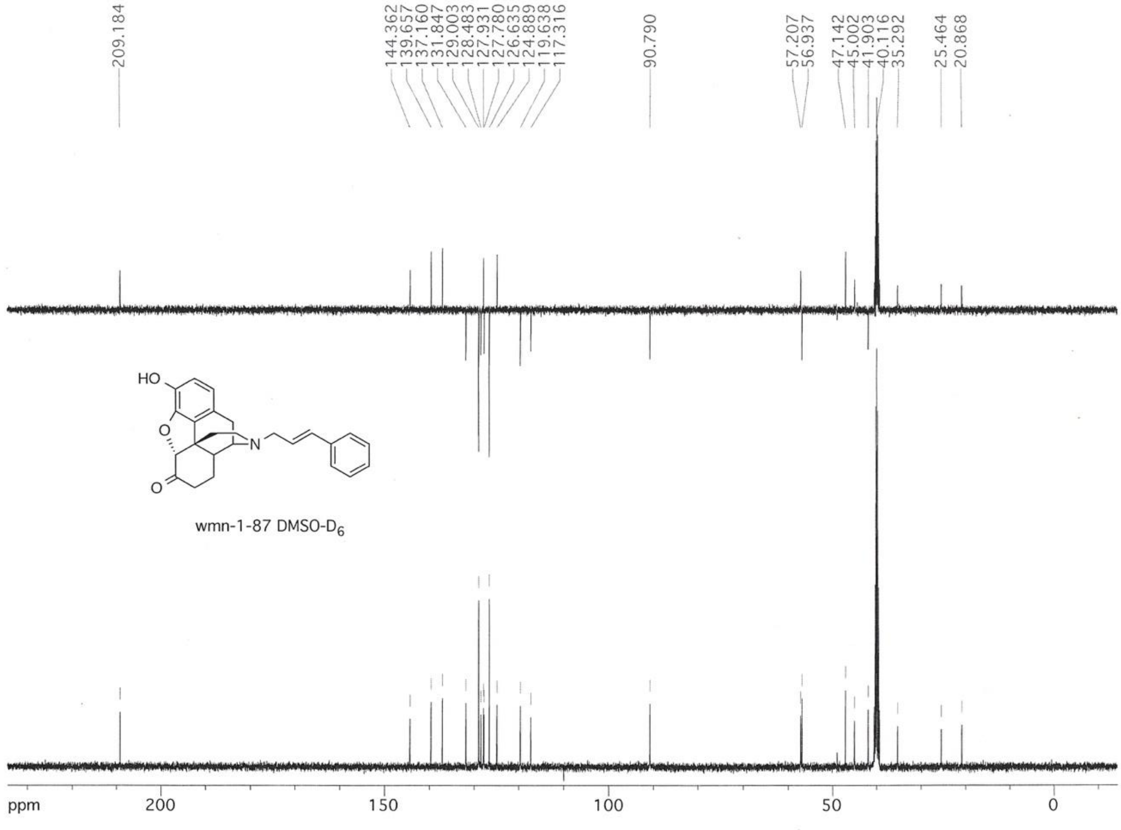

^{1}H and ^{13}C NMR of **1d**

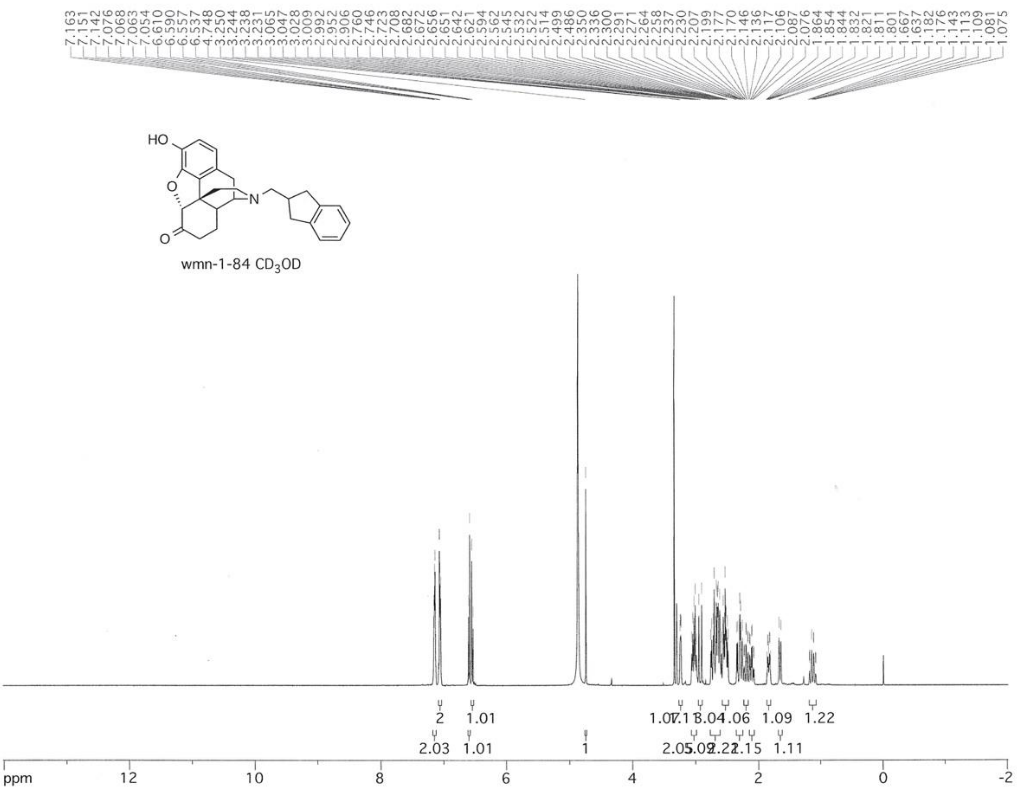

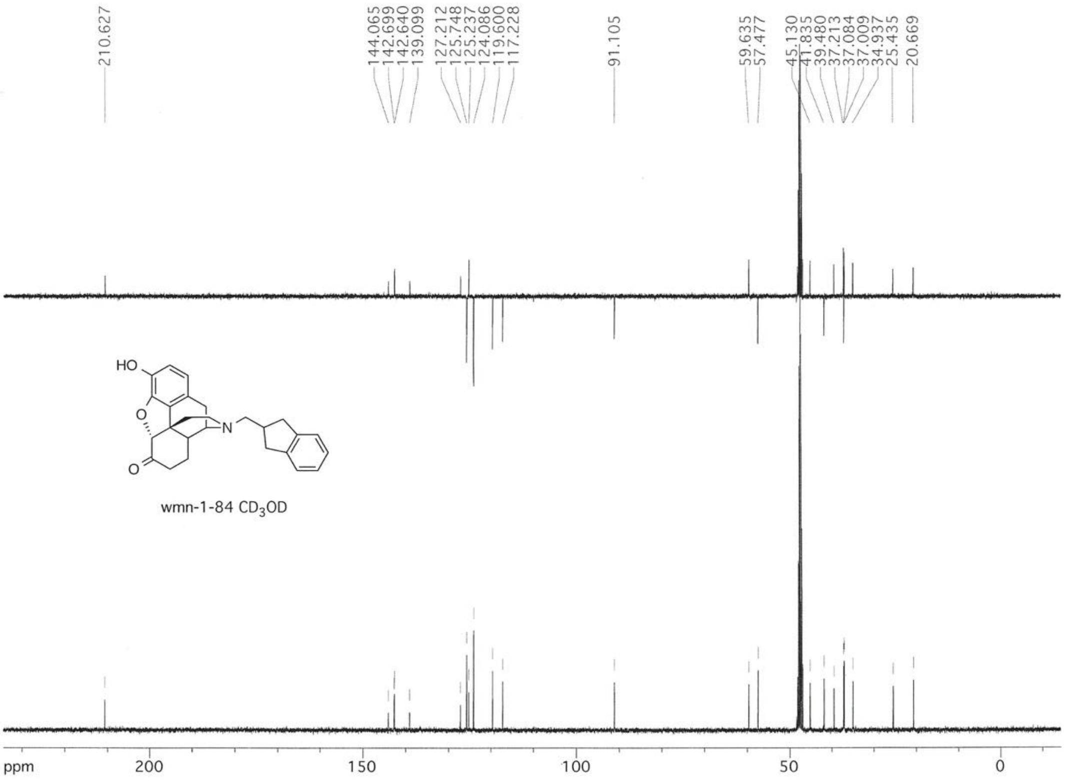

^{1}H and ^{13}C NMR of **2a**

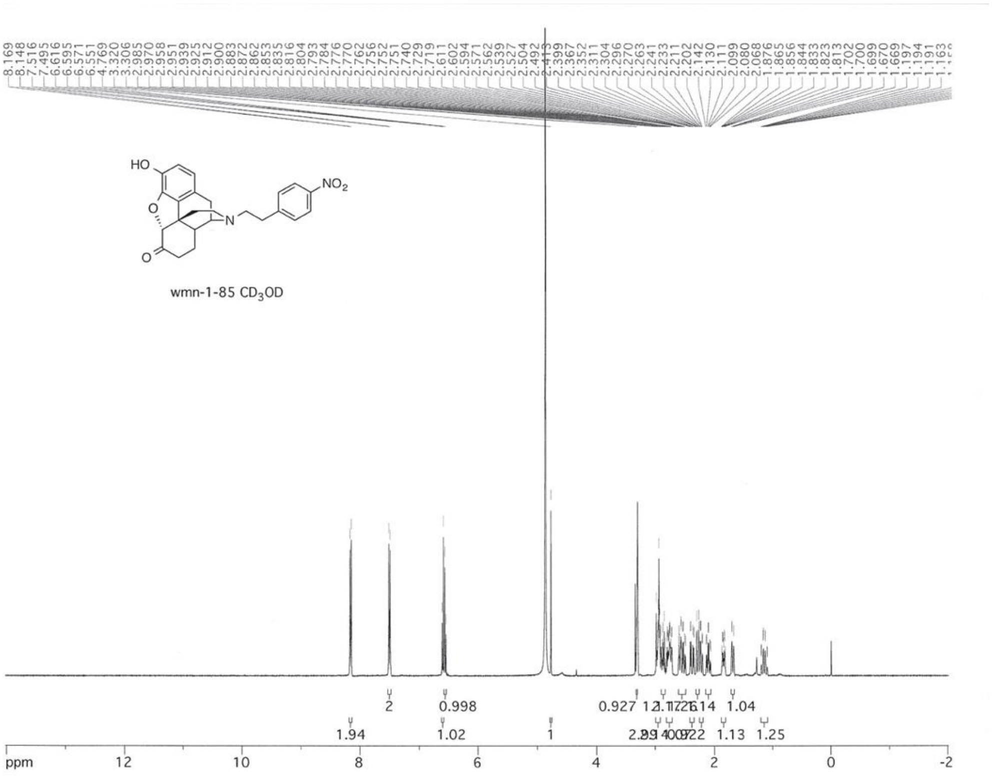

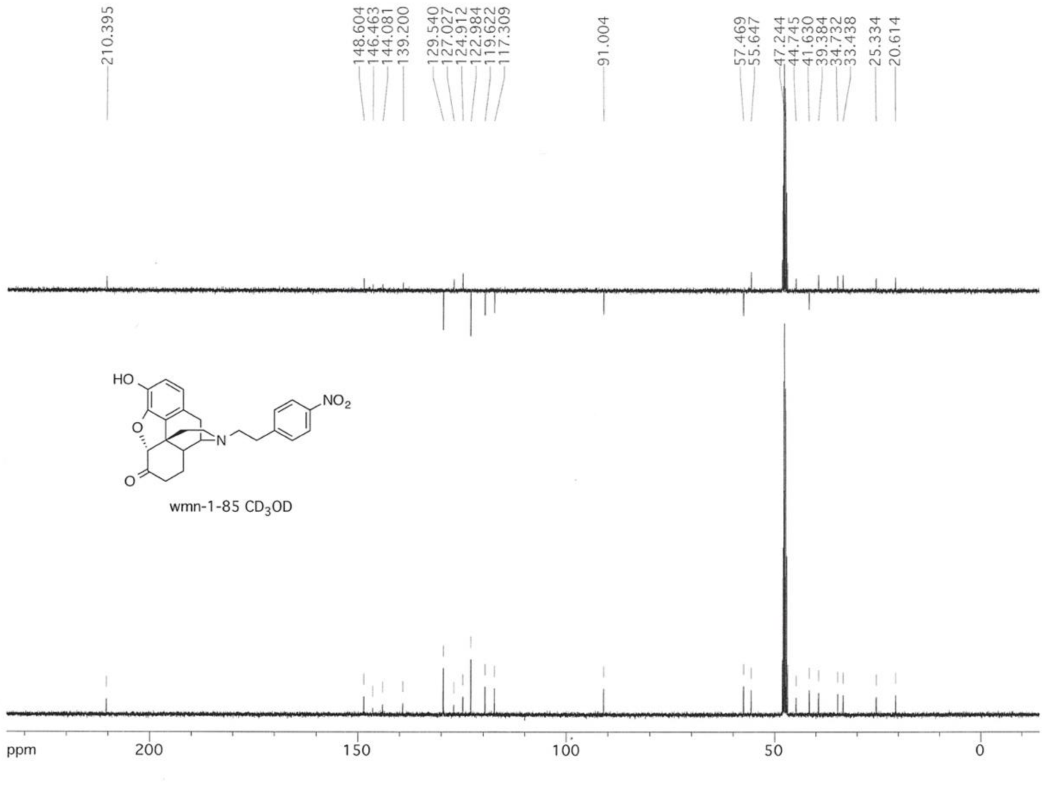

^{1}H and ^{13}C NMR of **2b**

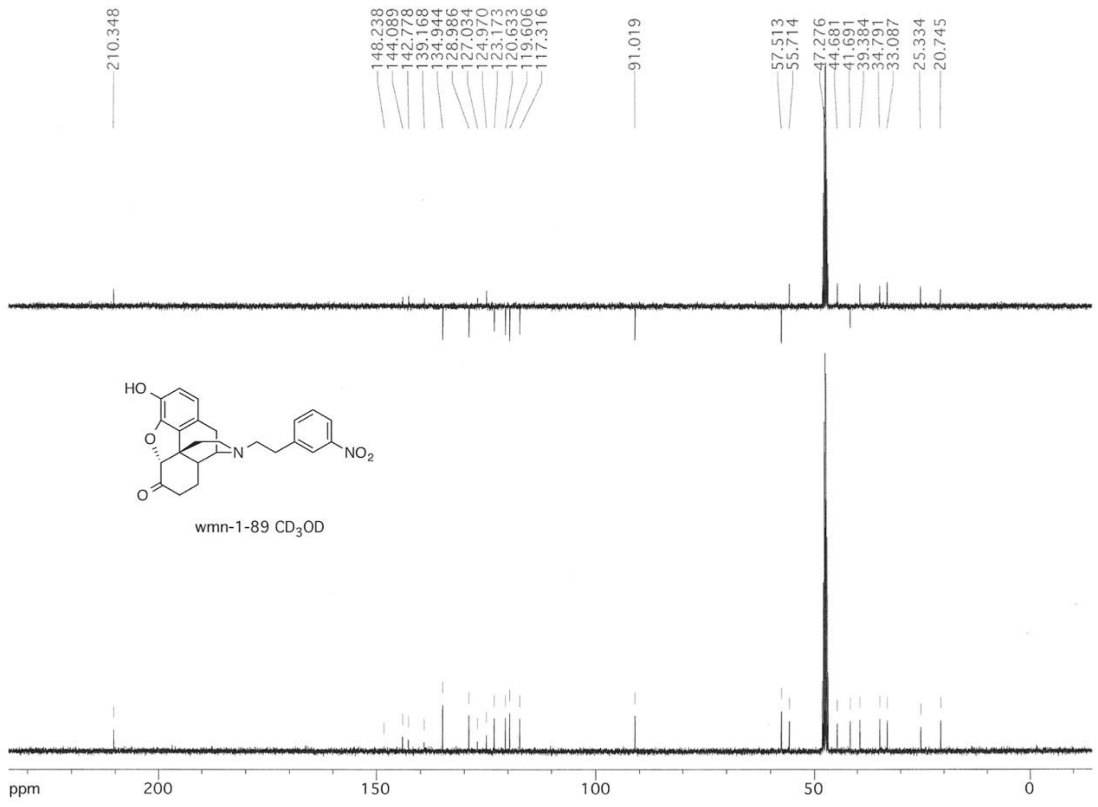

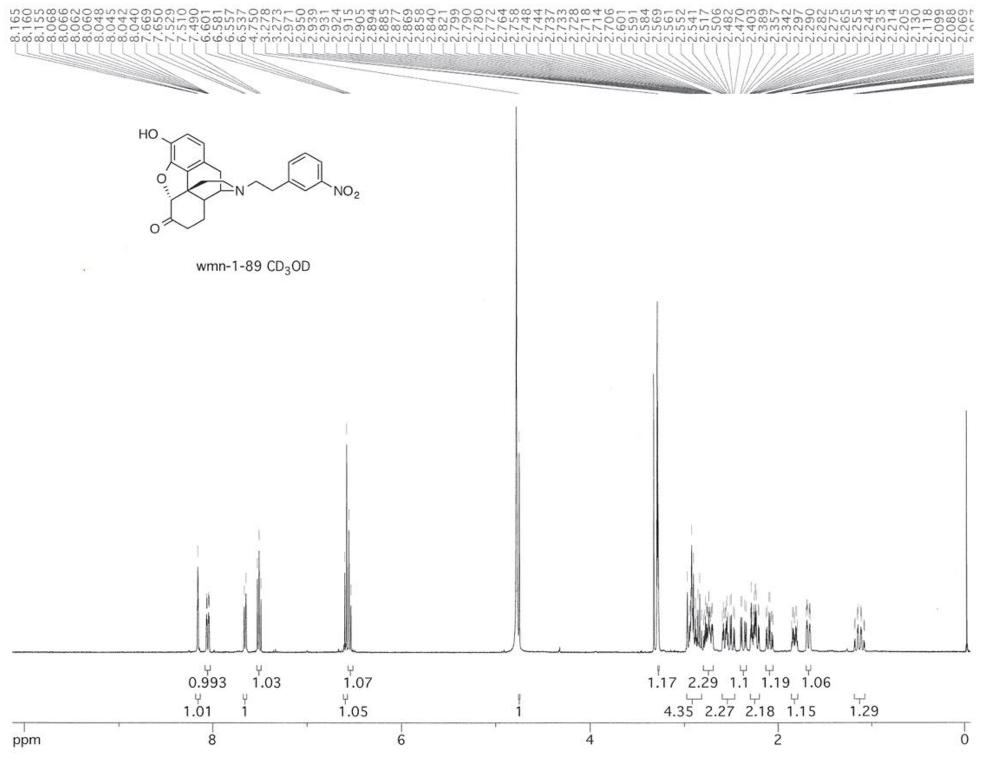

^{1}H and ^{13}C NMR of **2c**

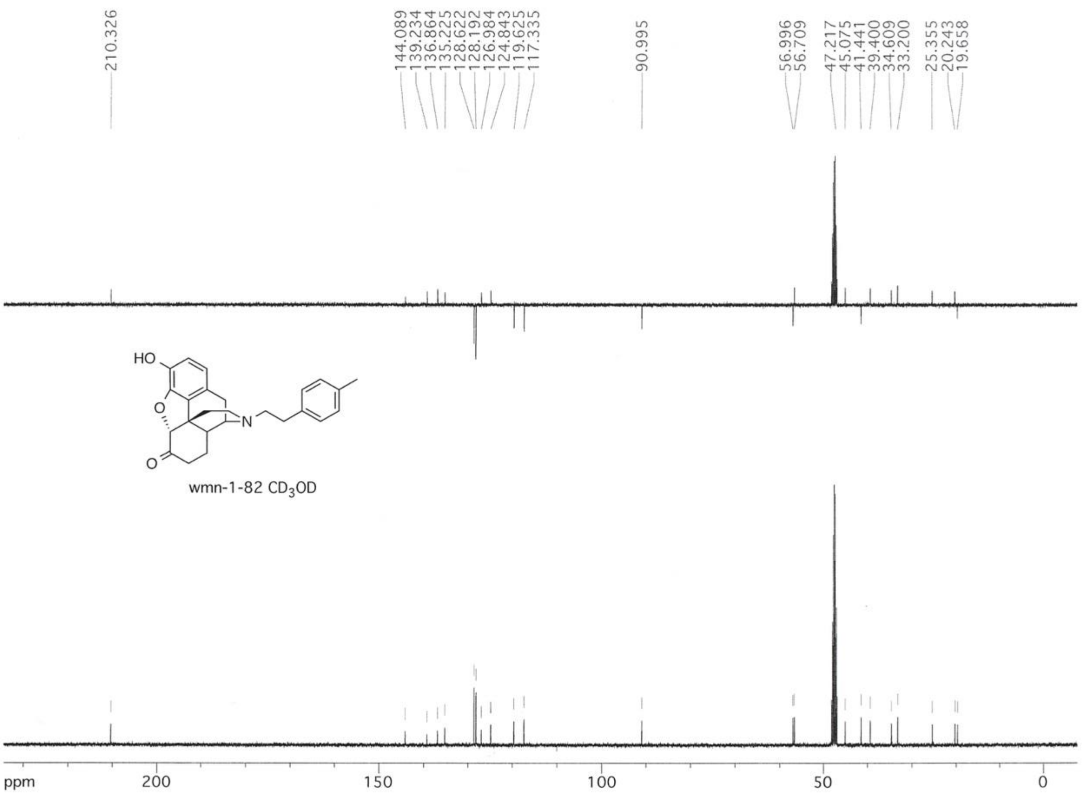

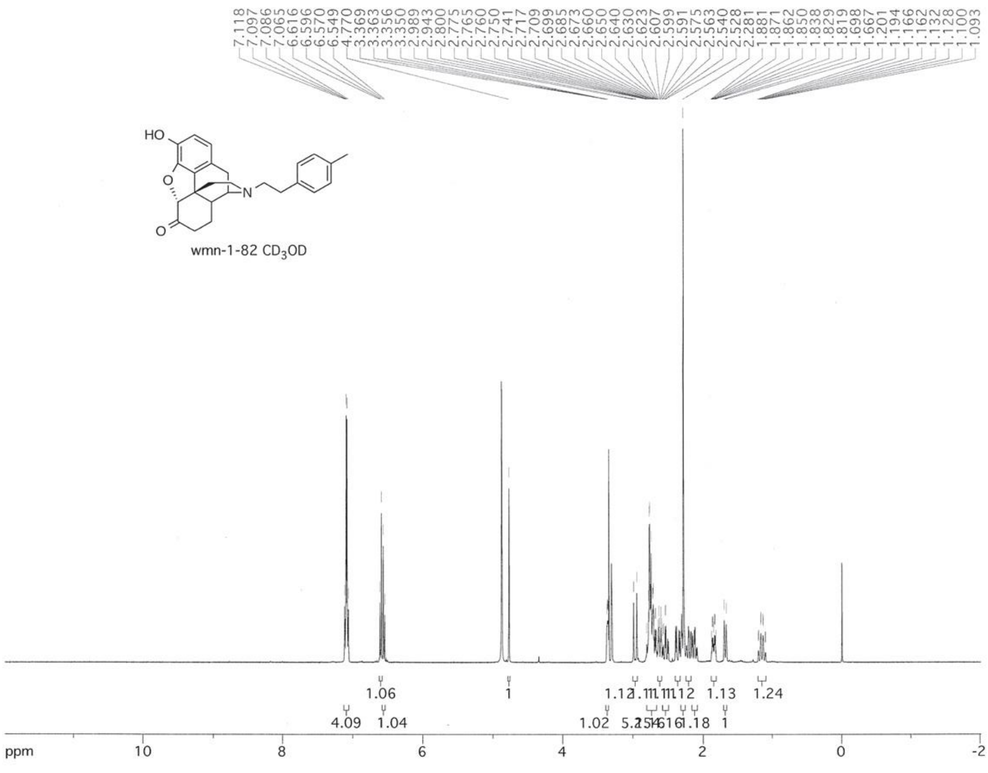

^{1}H and ^{13}C NMR of **2d**

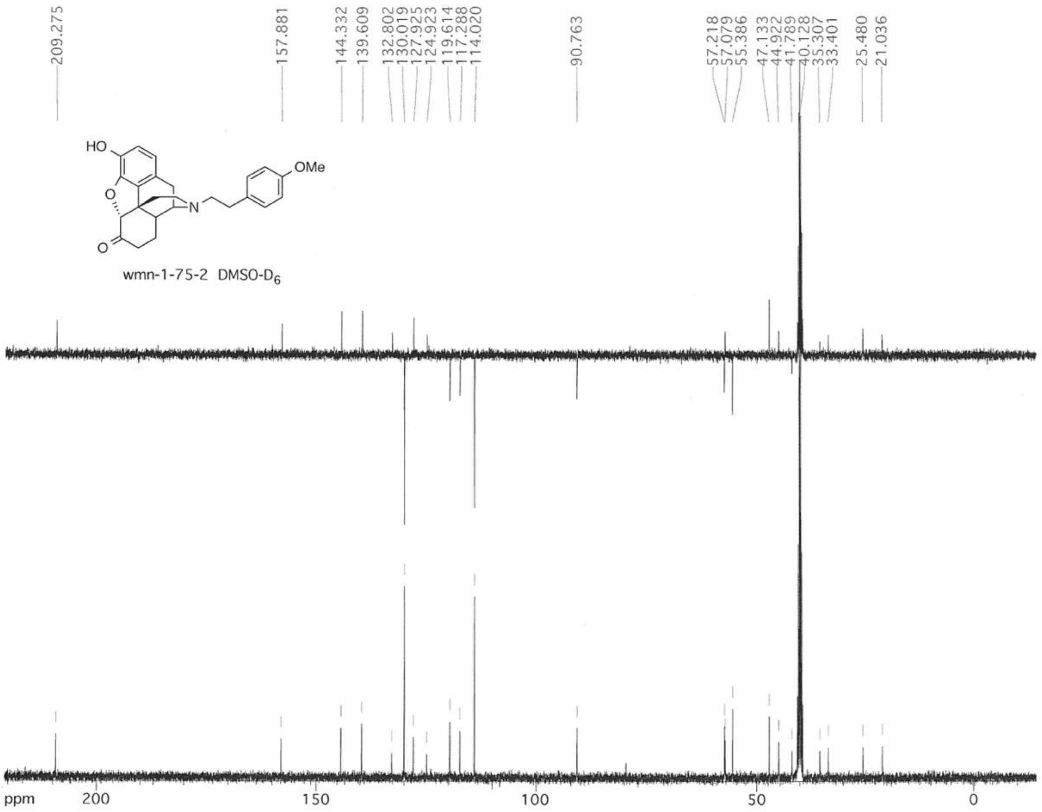

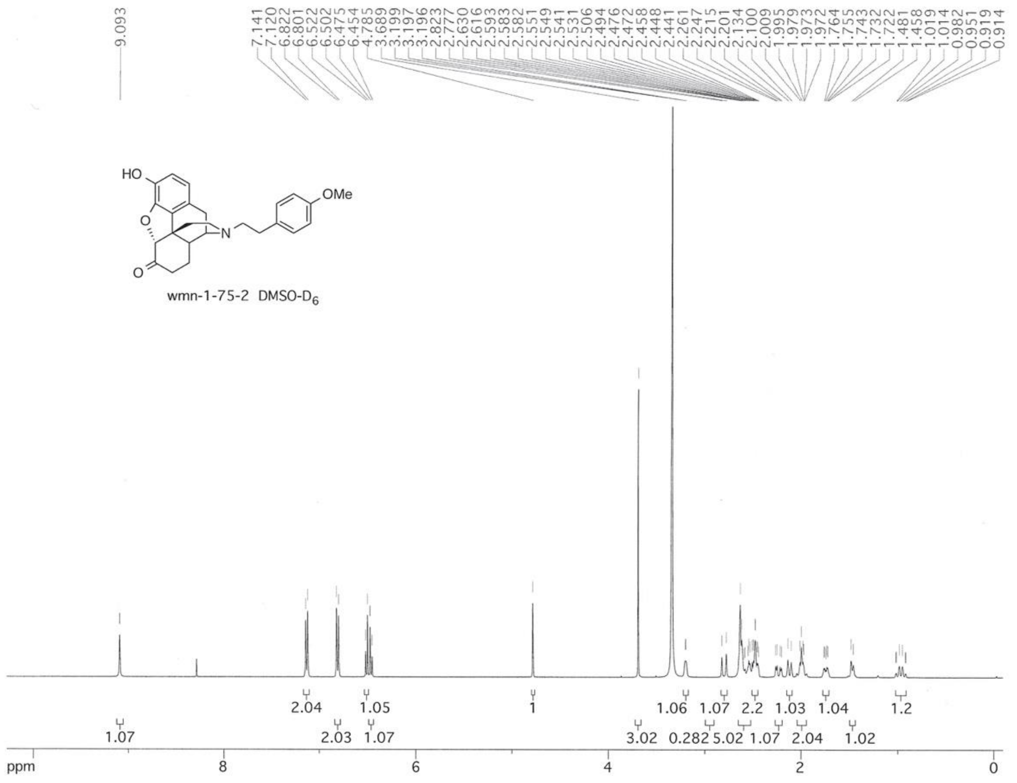

^{1}H and ^{13}C NMR of **2e**

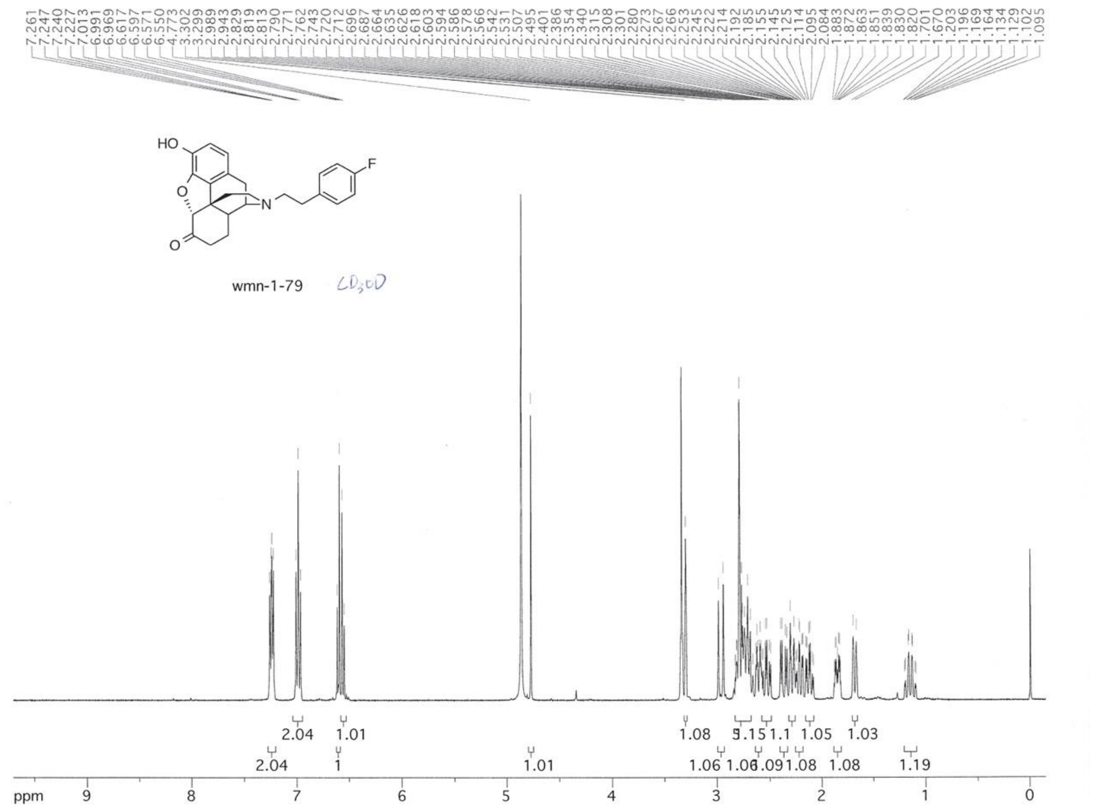

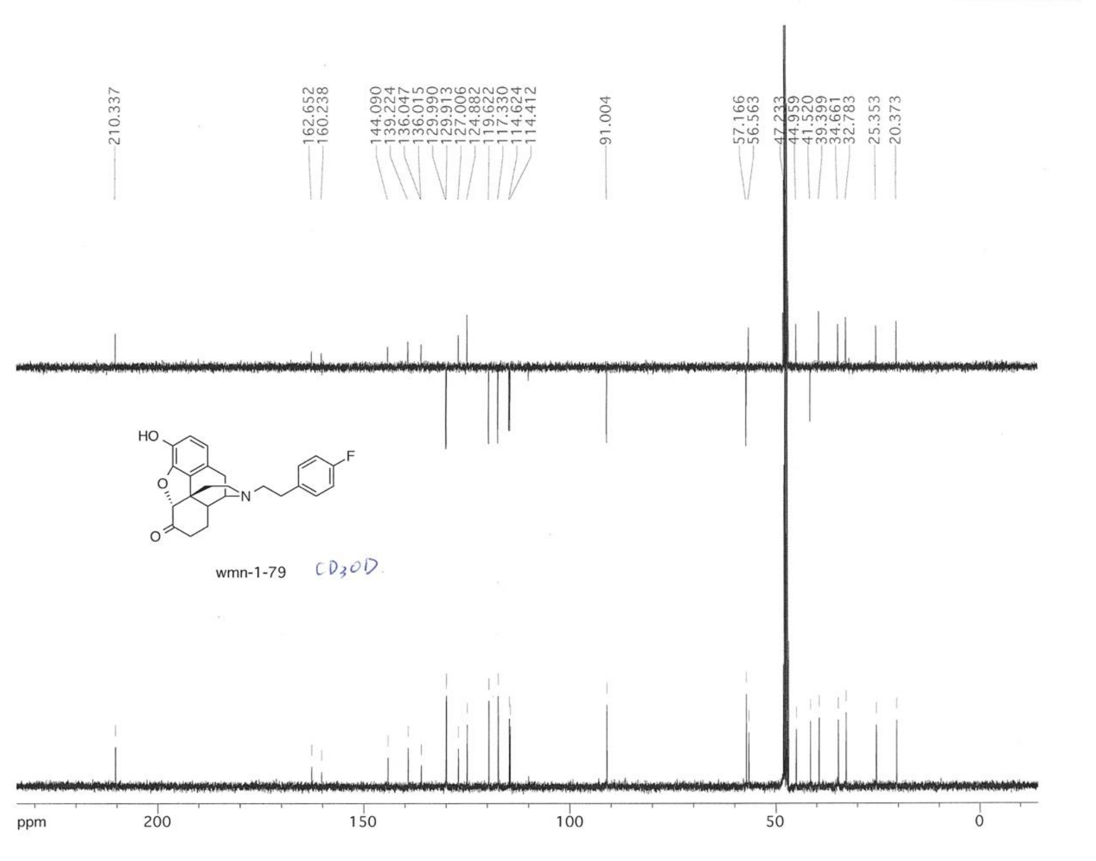

^{1}H and ^{13}C NMR of **2f**

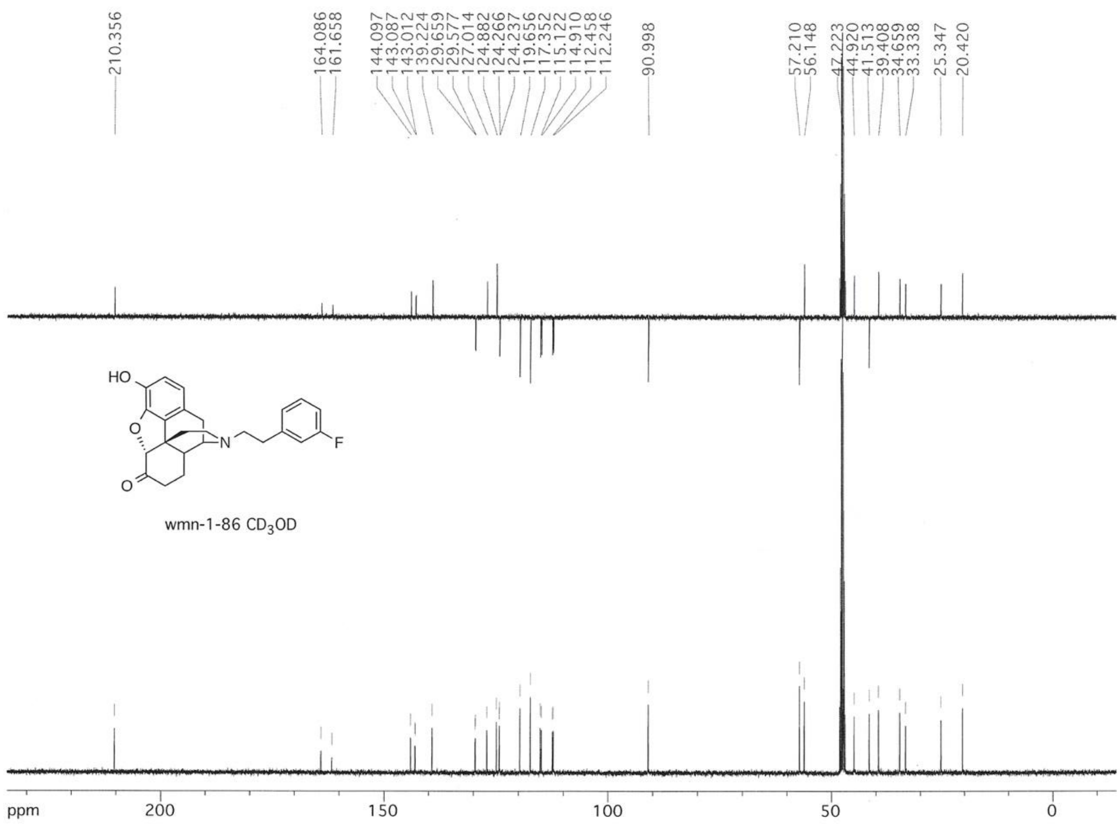

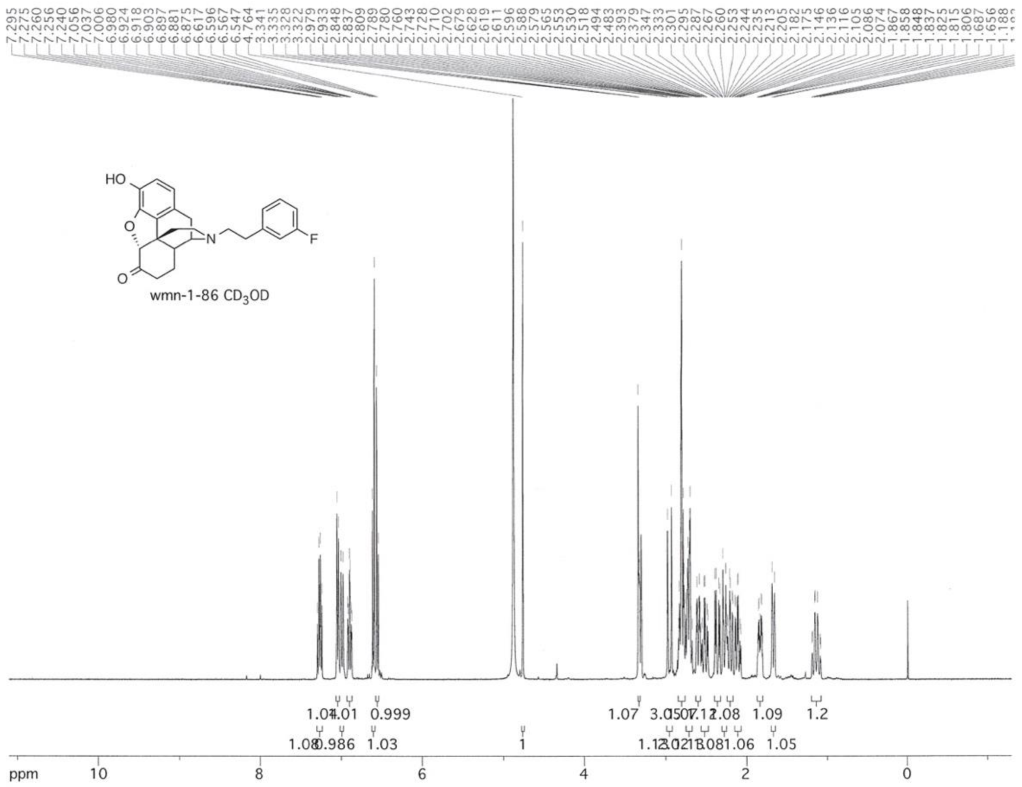

^{1}H and ^{13}C NMR of **2g**

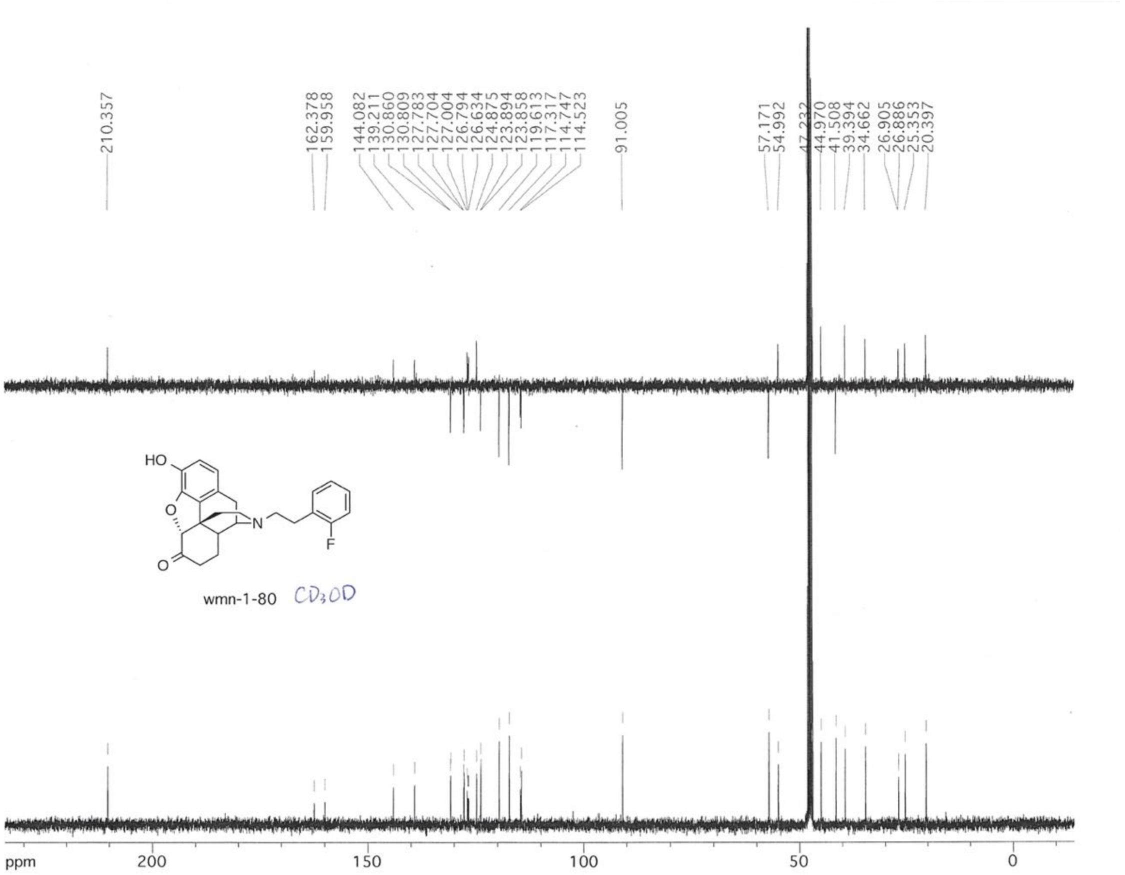

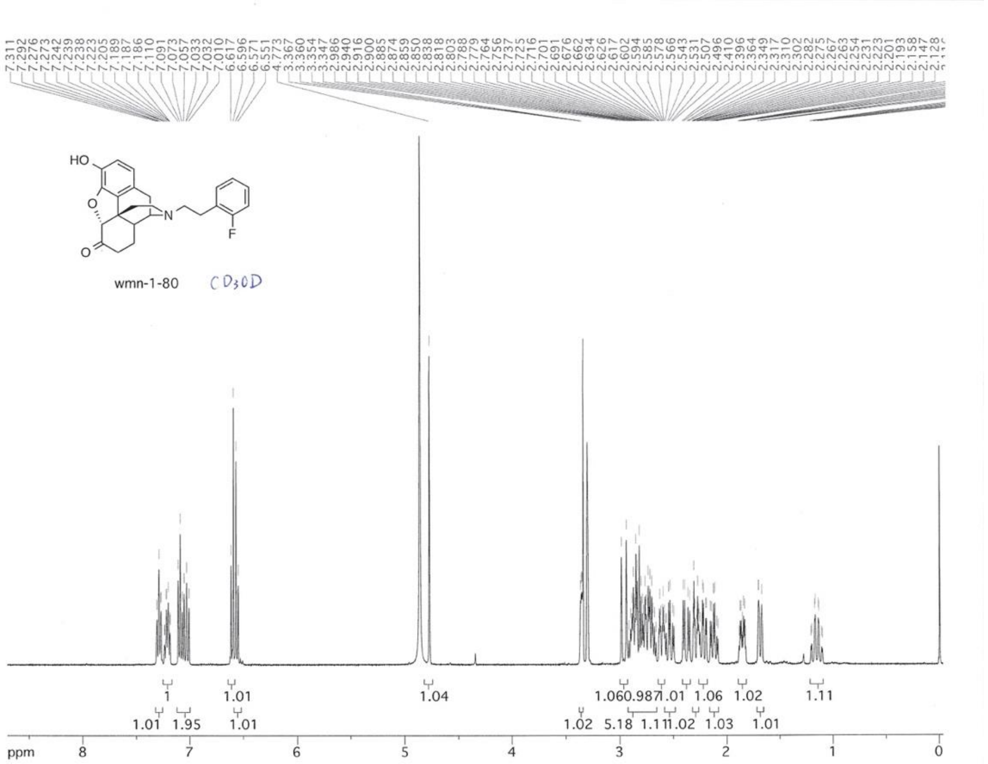

^{1}H and ^{13}C NMR of **2h**

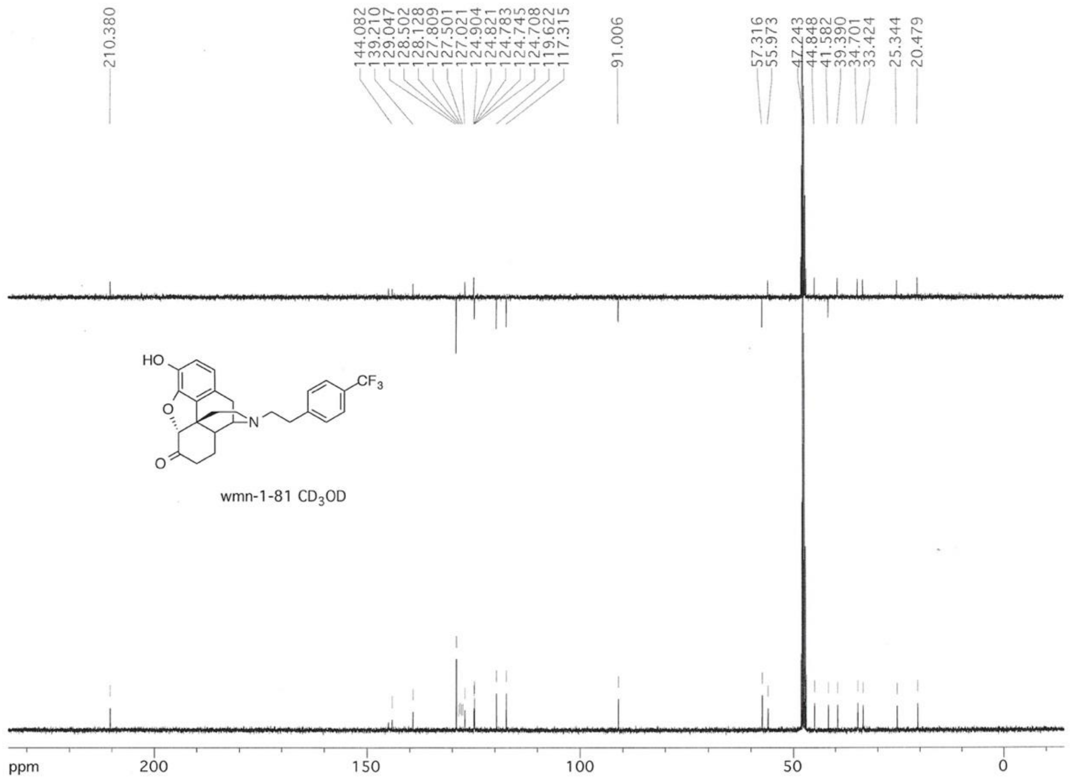

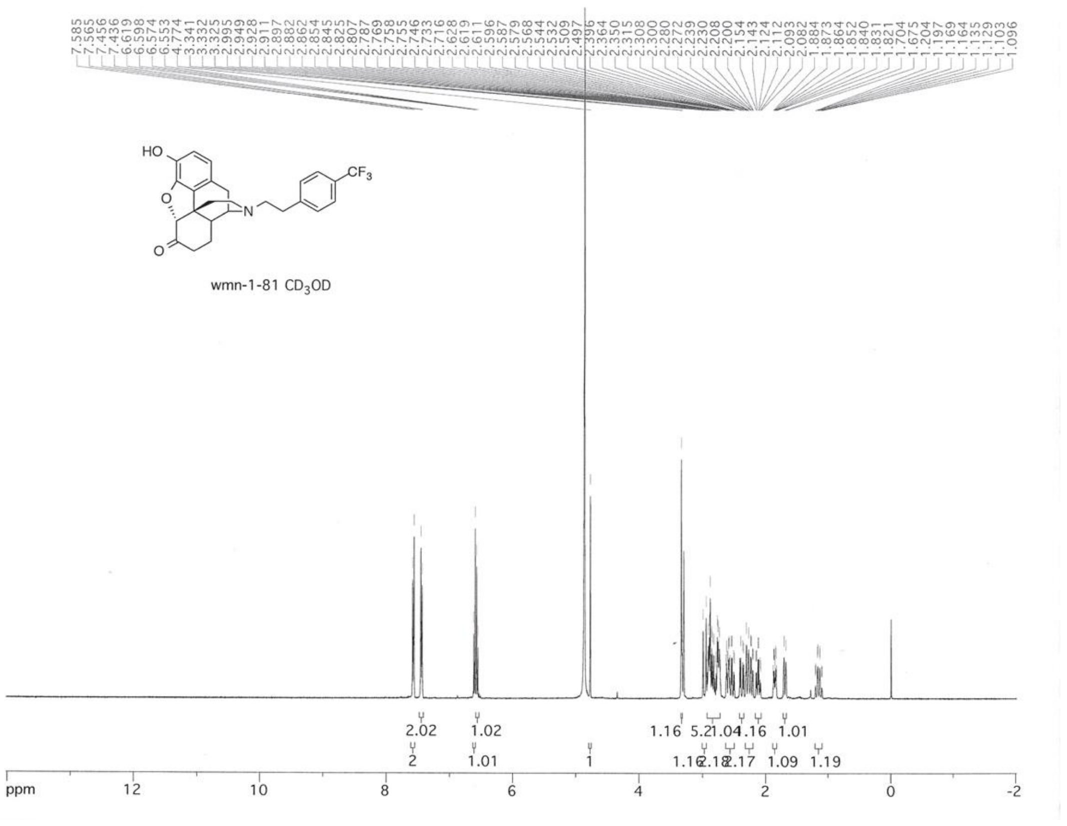

^{1}H and ^{13}C NMR of **2i**

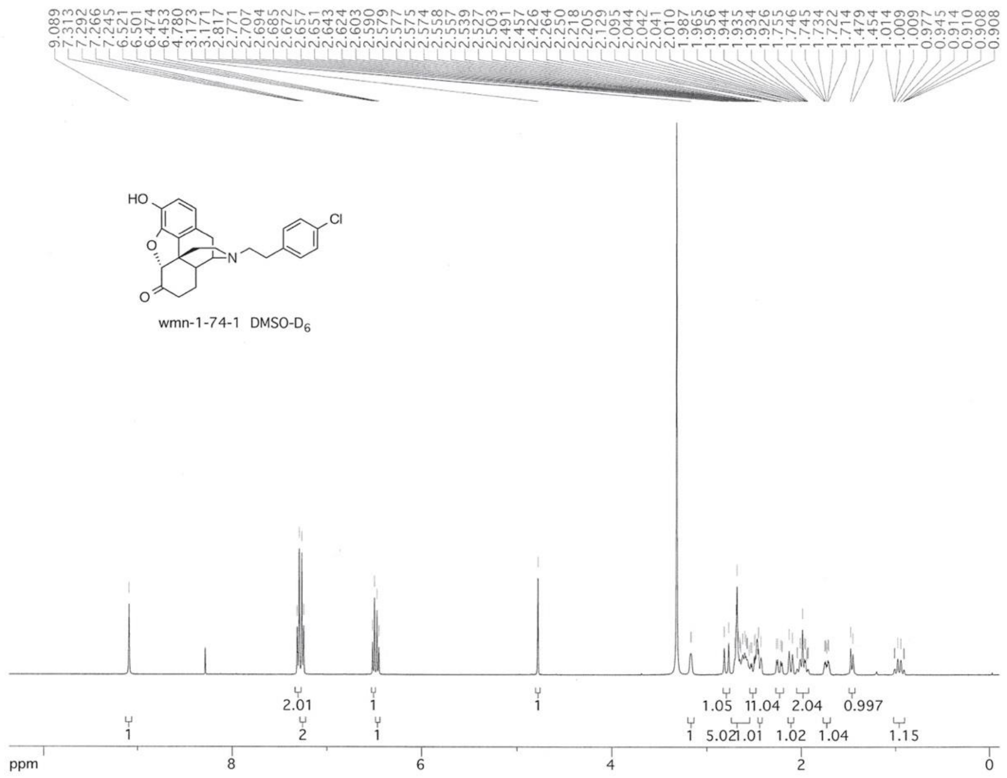

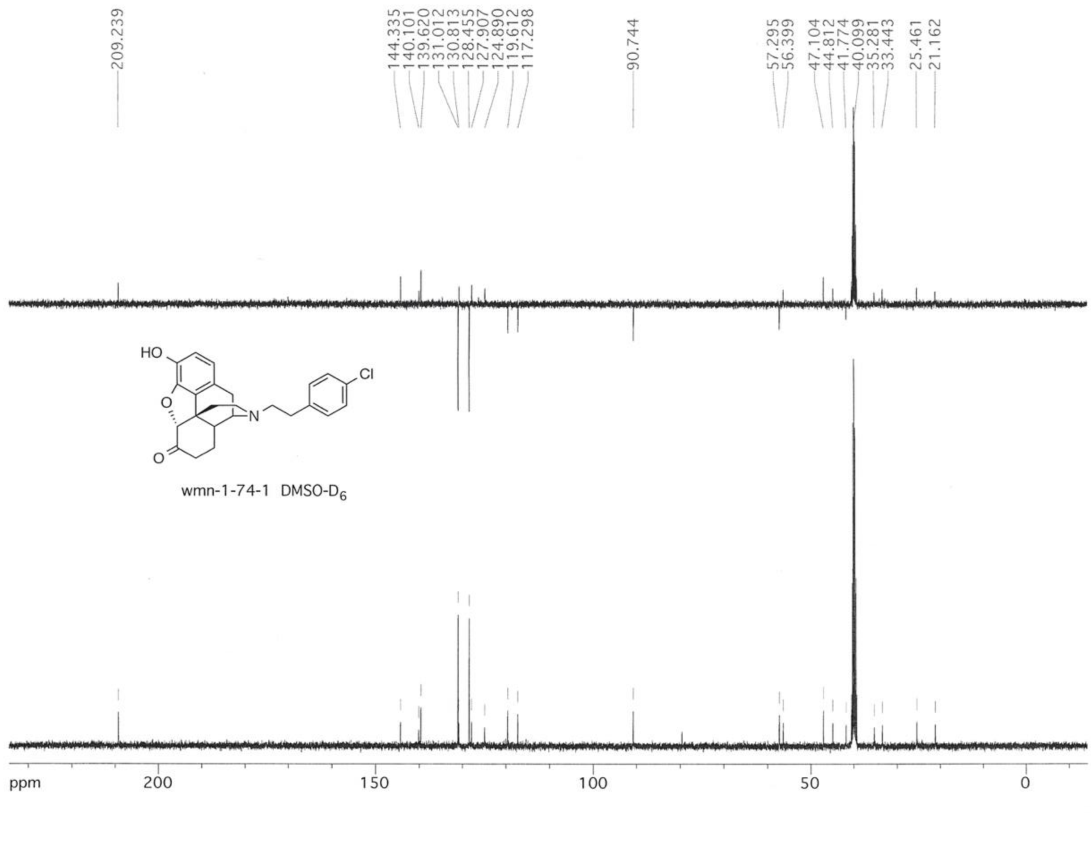

^{1}H and ^{13}C NMR of **2j**

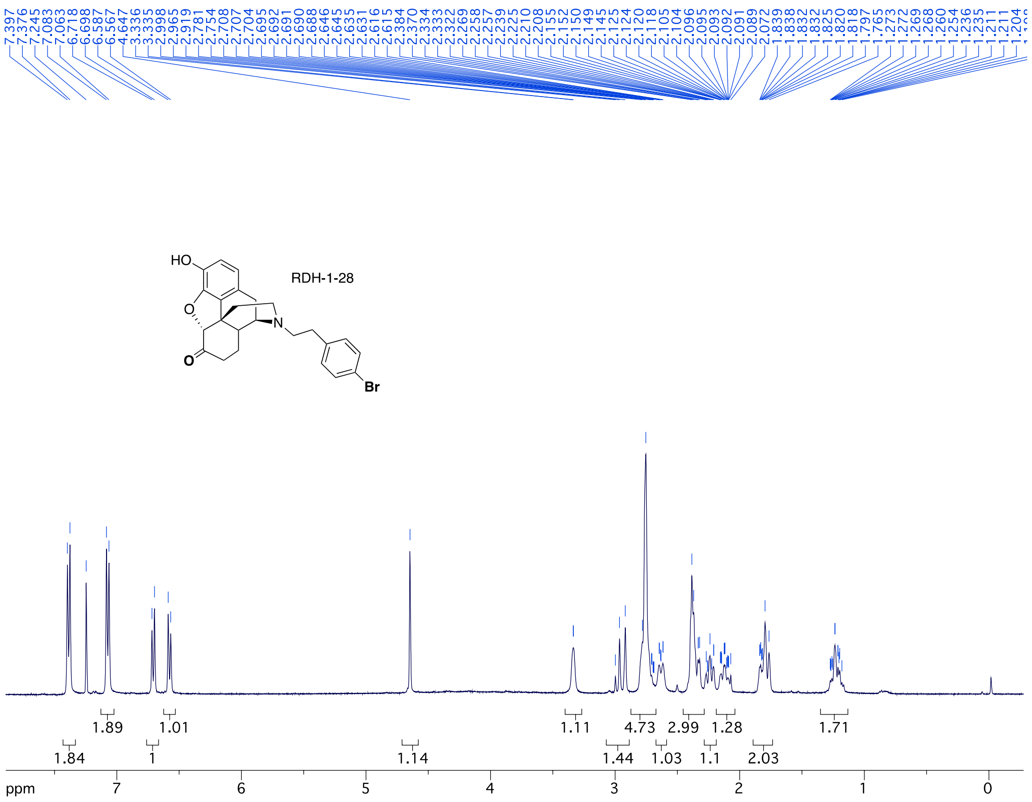

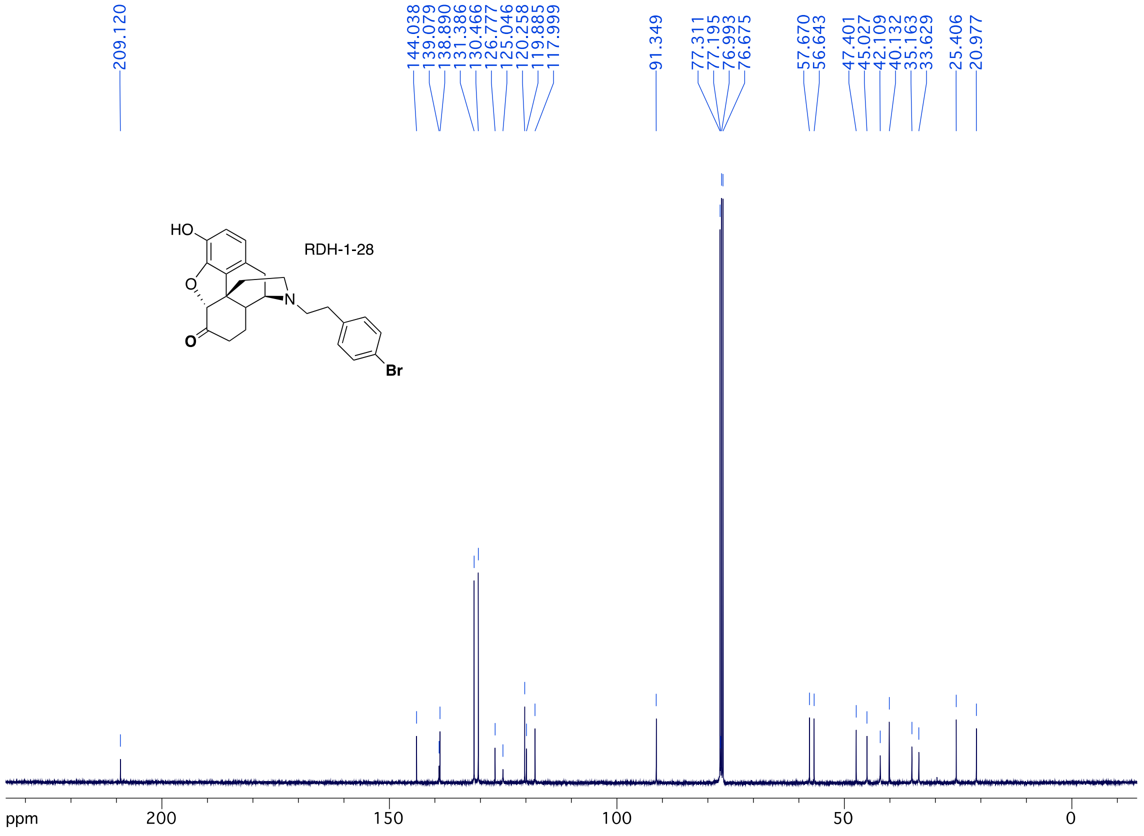

^{1}H and ^{13}C NMR of **2k**

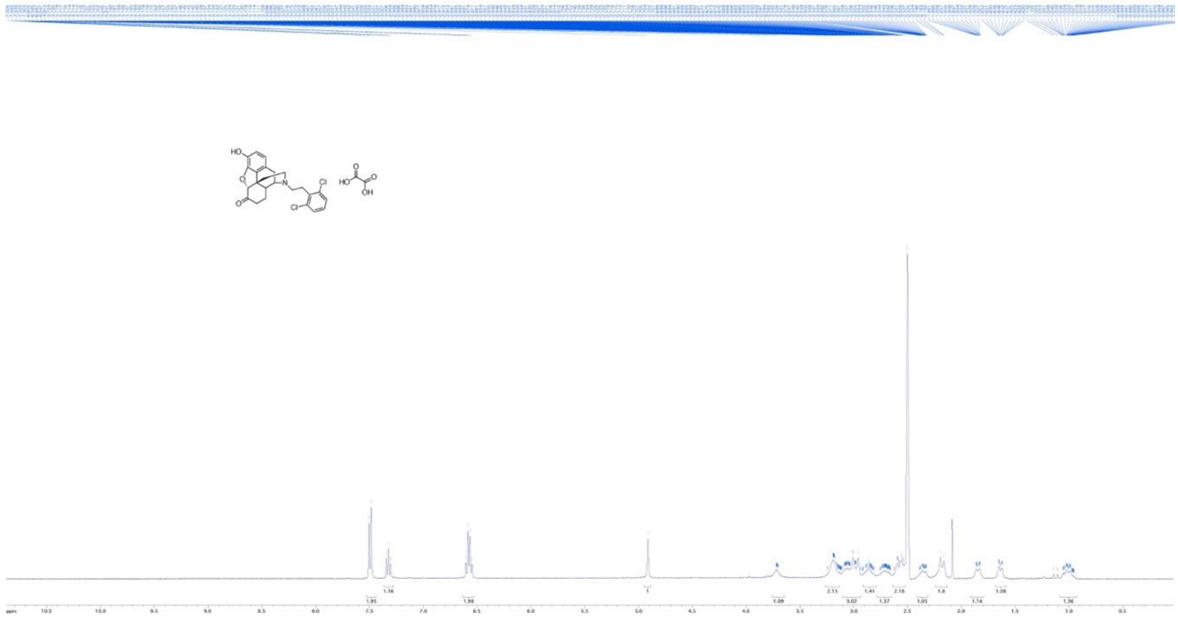

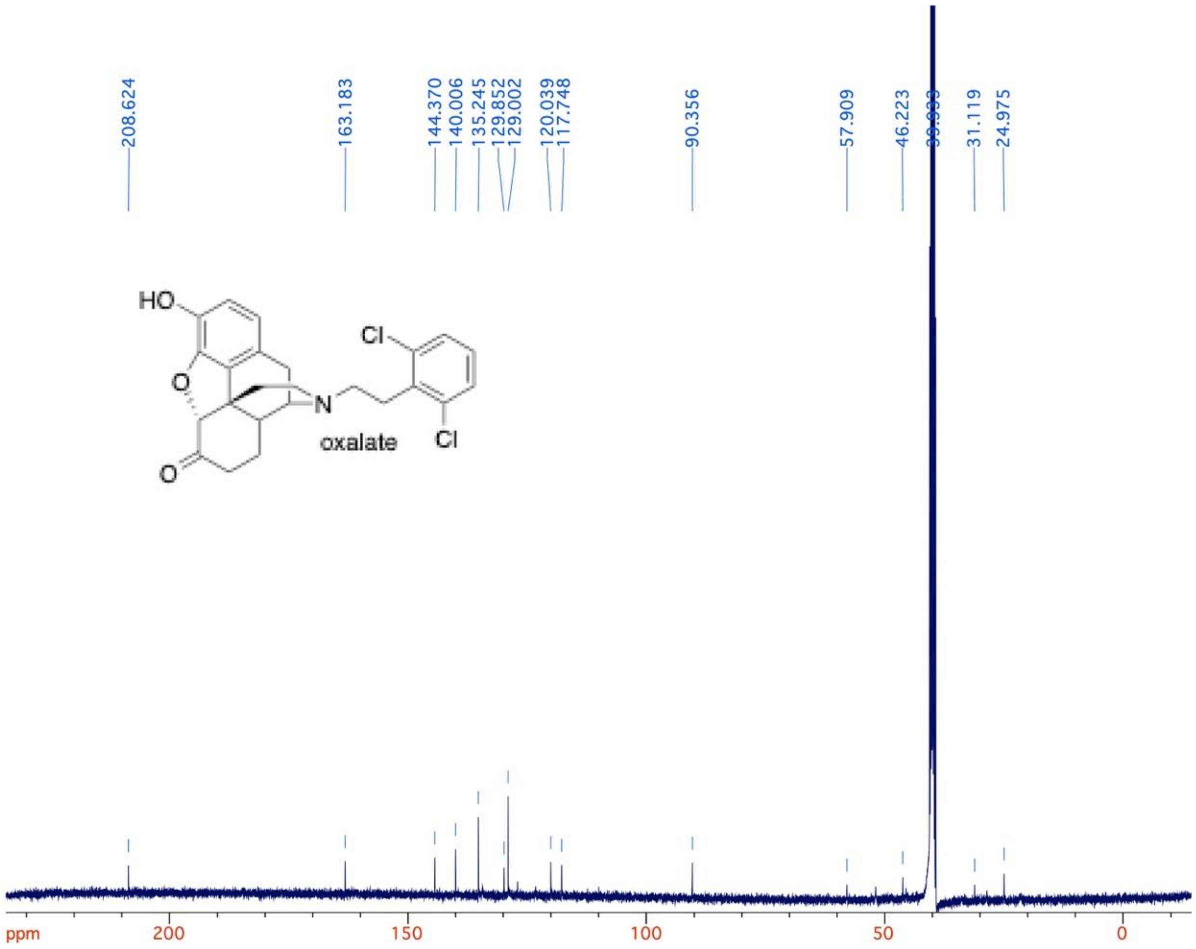

^{1}H and ^{13}C NMR of **S11** (*N*-Phenethylnorhydromorphone)

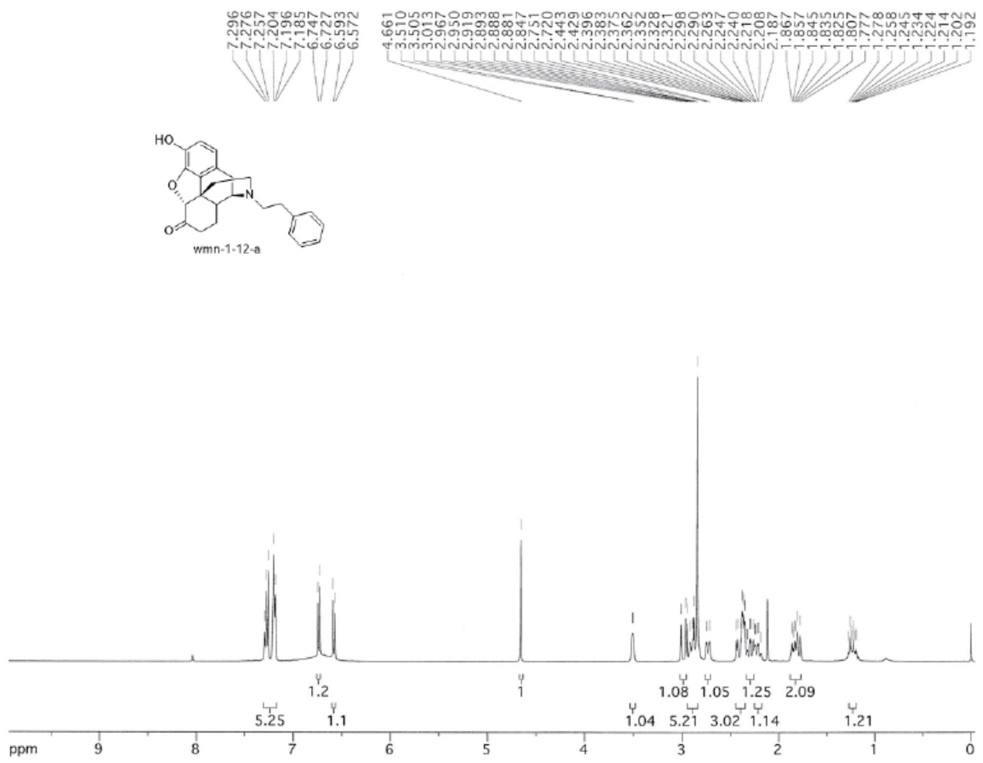

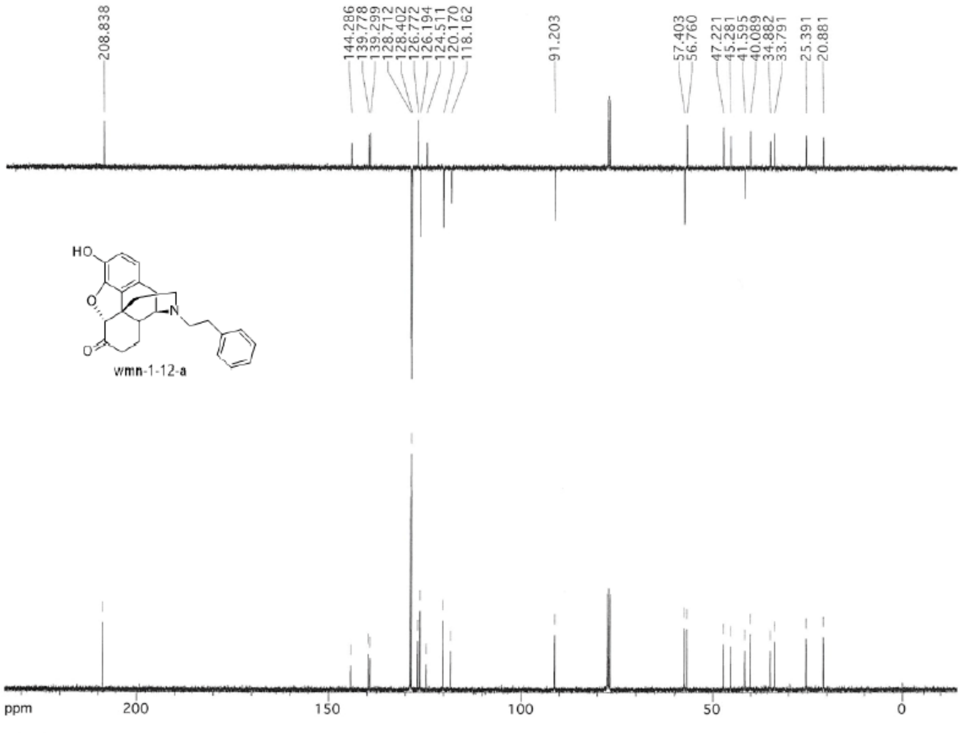

^{1}H NMR of **S5** (Hydromorphone)

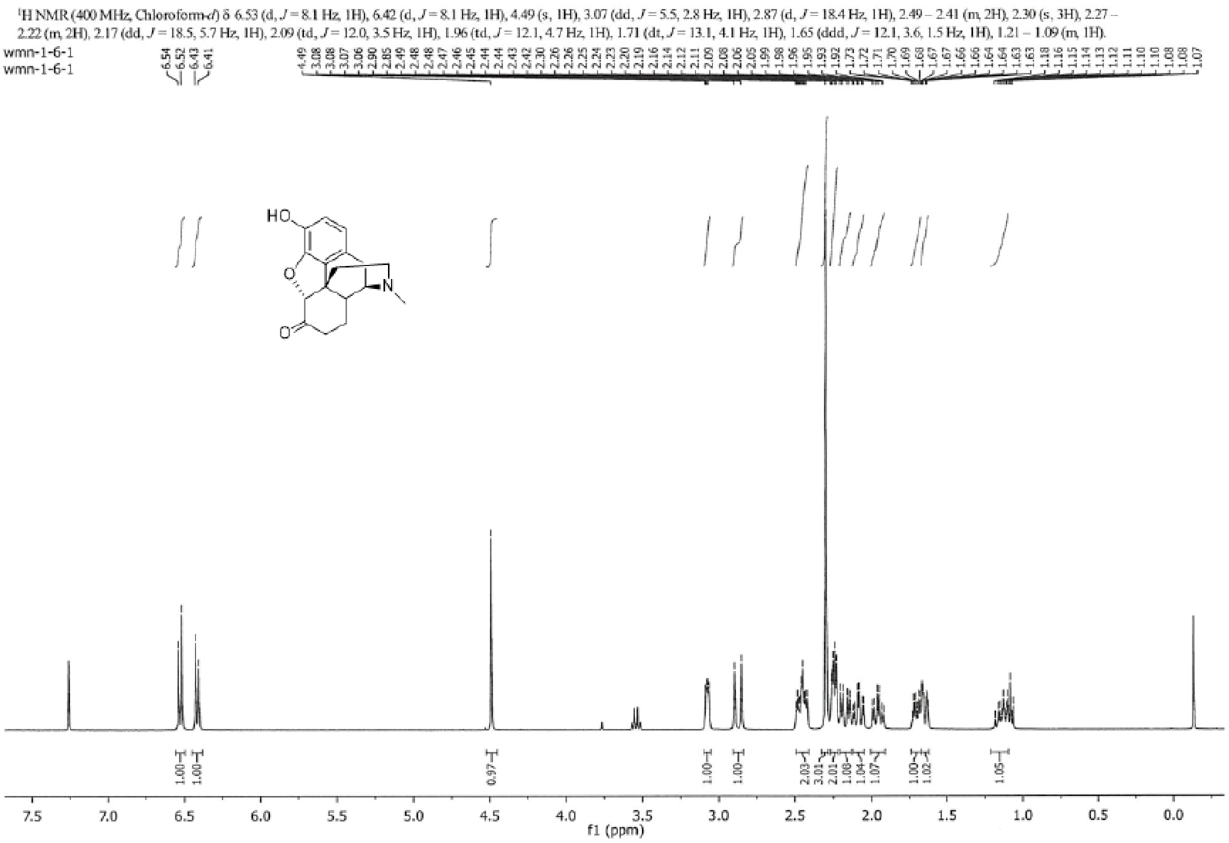

Table S1: Ligands studied by QM calculations and MD simulations [a]

Compound	Structure	μ	δ	μ % stimulation (EC50, nM)	δ % stimulation (EC50, nM)
**2g**	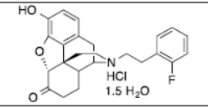	0.32 ± 0.04	3.57 ± 0.49	100 ± 0.3 (2.1 ± 0.7)<br>Potent full agonist	83.0 ± 3.3 (92.7 ± 17.0)
**2f**	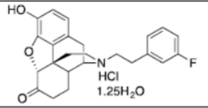	0.49 ± 0.07	4.48 ± 0.43	56.8 ± 7.5 (2.4 ± 0.3)<br>Potent partial agonist	54.1 ± 4.7 (86.7 ± 8.0)
**2e**	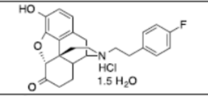	0.87 ± 0.10	13.4 ± 0.9	42.6 ± 2.9 (2.5 ± 0.3)<br>Potent partial agonist	64.2 ± 9.9 (243 ± 49)<br>Weak partial agonist
**2a**	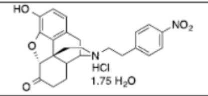	0.52 ± 0.01	6.07 ± 0.86	26.6 ± 1.4 (1.9 ± 0.5)<br>Potent partial agonist	64.3 ± 3.3 (227 ± 53)
**2b**	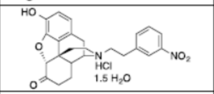	5.88 ± 0.38	92.5 ± .3	DNS<br>Antagonist	NT
**2i**	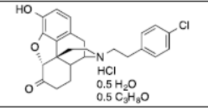	1.09 ± 0.06	7.89 ± 1.10	39.0 ± 2.1 (2.0 ± 1.0)<br>Potent partial agonist	83.8 ± 3.7 (2.4 ± 0.7)<br>Potent full agonist
**2d**	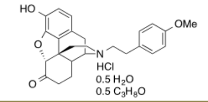	0.67 ± 0.05	5.43 ± 0.47	DNS<br>Antagonist	48.9 ± 3.2 (33.6 ± 6.4)
**S11**	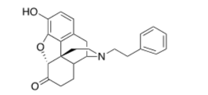	NT	NT	100 (0.237 ± 0.09)<br>Potent full agonist	96 (21.89 ± 0.87)<br>Weak full agonist

[a)] MD simulations of the MOR and DORs were carried out for the eight ligands shown, each with multiple *N*-phenethyl group conformers (Fig. S2). Comparative analysis of the simulations, along with the experimental data, allowed us to propose a tentative set of rules to design ligands with specific properties. The colored boxes indicate independent comparative analyses: dark blue (**2g**, **2f**, **2e** in the μ column): effect of the fluorine position in the ring; green (**2a**, **2b**, μ column): effect of the nitro position; red (**2e**, **2a**, **2i**, **2d**, μ column): effects of chemical substitutions at the *para* position; light blue (**2e**, **2i** in the δ column): effect of F/Cl at the *para* position.

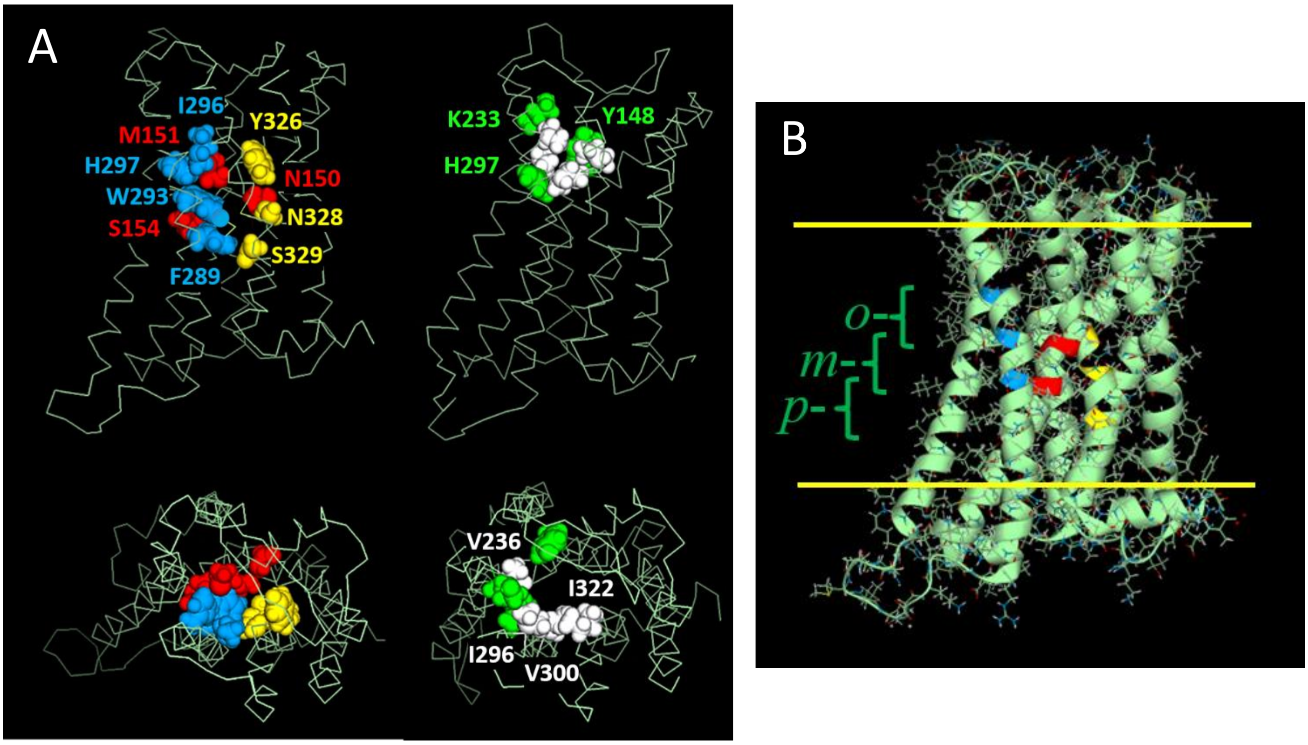

Figure S1: (A) left panel: critical residues that interact with the substituent groups in the *N*-phenethyl ring of all the ligands in the series (**Table S1**) are limited in number and located at specific positions in only three TMH (Sequence numbering as in MOR; same positions in the 3D structure for DOR; cf. Figs. 1, 2, and S3): residues on TMH 3 are colored red, TMH 6 in blue, and TMH 7 in yellow; right panel: residues that interact with the body regardless of *N*-phenethyl ring substitution: polar/charged residues in green, nonpolar in white. (B) When substituents are moved around the ring (indicated by *o*-, *m*- and *p*-), they target different TMHs and at different levels of the receptors within the transmembrane region (colors as in left panel of A); membrane boundaries indicated by yellow lines.

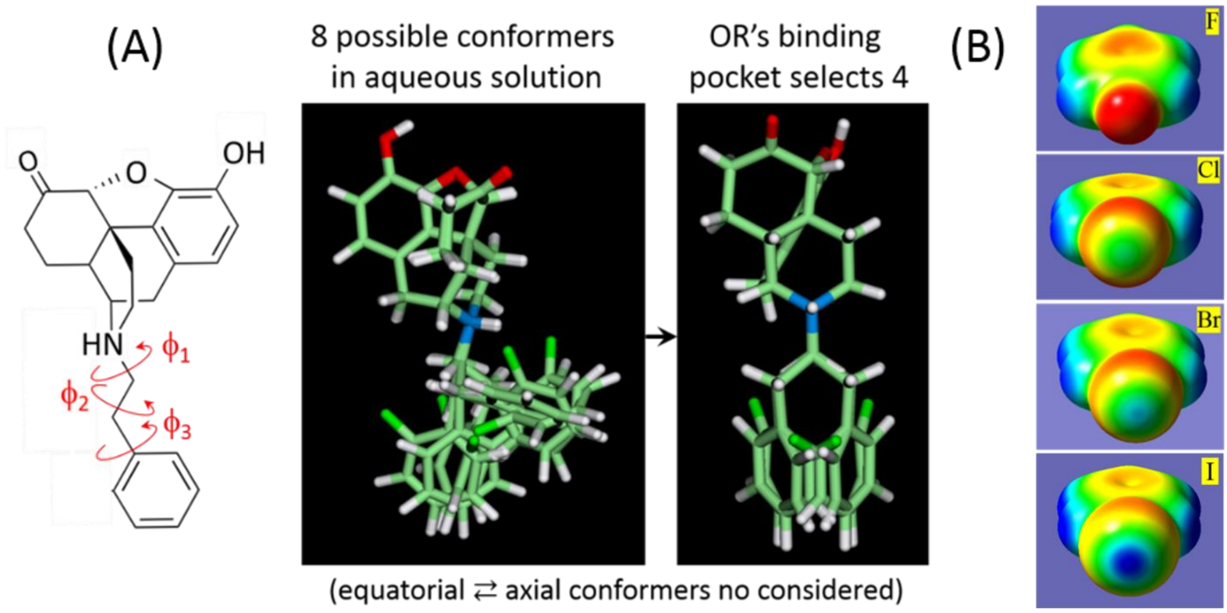

Figure S2. (A) Possible degrees of freedom of the N-phenethylnorhydromorphone molecule in an aqueous solution. There are eight isoenergetic conformers within ~1 kcal/mol; steric clashes with residues in the pocket select up to four of these conformers; the dihedral ϕ_3 is important for *m* and *o*- substituted molecules; methoxy substituted ring can add yet another rotation around the O-Me bond. All the selected conformers appear to be well stabilized, either by hydrogen or halogen bonding between the substituent group and specific residues lining the pocket (cf. Fig. 1), and by the same nonpolar interactions that stabilize the ring in the parent (unsubstituted) compound. (B) Electrostatic potentials mapped on the isodensity contour at 0.001 e/bohr^3 for fluorobenzene, chlorobenzene, bromobenzene, and iodobenzene. These illustrate the electron density distribution, including the σ-hole and equatorial negative field on Cl, Br, and I which may affect the interactions with residues lining the OR binding pocket (see text). Utilizing the density functional theory at the level of B3LYP/DGDZVP (Gaussian 09 [46]), the structures were first geometry optimized in the gaseous phase and their electrostatic potential maps then constructed.

A comparative analysis of the simulations data allowed us to identify a reduced number of residues that interact with the body and tail of the ligands and which are responsible for the differences in receptor properties. Together, the experimental and simulation data suggest a set of rules that the ligands need to obey to induce specific behaviors: **i)** Agonism follows from the tail engaging TMH 3 and 6, or 2 and 7, or (not too strongly) 6 and 7; antagonism, when the tail engages TMH 3 and 7 or (strongly) 6 and 7, or when it interacts with a single TMH, either 2, 3, 6 or 7. **ii)** Partial vs. full agonist properties of a ligand correlate with the number of conformers with predicted agonist activity. Full agonism may thus be enforced by restricting the number of inactive conformers by making the tail more rigid. Alternatively, multiple substates of the receptors for a given conformer may also lead to partial agonism if the OR is locked in inactive substates; but we did not detect multiple, stable receptor conformations within the time scale of the simulations. **iii)** Relative affinities appear to correlate with the number and frequency of favorable contacts (polar/nonpolar, H-/halogen-bonding) between the substituent group and the OR; other contacts (body- and phenethyl ring-OR) are common to all the ligands/conformers and thus less critical in modulating the OR behavior.

The residues identified in the simulations (Fig. 2 and Fig. S3) are shown in their structural context in Fig. S1 and should be the target of systematic SAR synthetic efforts since they alone are expected to control and modulate the behavior of MOR and DOR independently. Because the plane of the *N*-phenethyl ring is perpendicular to the membrane, small changes in the location of the substituent can target the ORs at different levels across the transmembrane region (Fig. S1B). The pattern of interactions is distinct in each level: *p*-substituents tend to interact simultaneously with several residues at the bottom of the pocket and have the potential to affect several TMHs; in the opposite limit, the *o*-substitutions tend to interact with fewer residues (e.g., *o*-F in Fig. S3C) or even a single residue in specific directions in the upper regions of the pocket, depending on the

conformation of the tail and chemical nature of the substitution; the *m*-substituents fall in between both. Regardless of the substituents, the body interacts, in varying degrees, with only three polar/charged residues and is further stabilized by hydrophobic contacts with four other nonpolar residues.

Table S2: Frequencies of contacts for stable conformers [a]

	2a	2b	2d	2e	2f	2g	2i	S11
MOR	85 95	100 95<br>90 70	55 95	50 100	75 85<br>85 95	100 100<br>100 100	70 100	85
DOR	100 100	100 100<br>100 100	100 100	100 100	100 100<br>100 100	100 100<br>100 100	100 100	100 80

[a] Frequencies of contacts between -NH of the ligands and the conserved D147 (MOR) or D128 (DOR) calculated for each stable conformer (between one and four out of eight for each ligand; cf. Fig. S2A) of the eight compounds in Table S1. Each conformer corresponds to a specific configuration of ϕ_1 and ϕ_3, and occupies a quadrants in each cell of the table (they appear in the same relative positions, so they are comparable across ligands and ORs); each entry indicates the percentage of simulation time the contact was observed, with the condition that $d < 3.5$ Å, where d is the distance between the protonated nitrogen of the ligand and the oxygen atoms of the Asp^- side chain. Blank entries mean that the frequency was lower than 50%, so the conformer was deemed unstable and discarded (see Methods in main text). The same quantitative analysis, with a different percentage criterion (25%) for H-bond/polar contacts, was performed for all the body-OR and tail-OR interactions shown in Figs. 1, 2, and S3. (Atomic coordinates of the conformers are available upon request.)

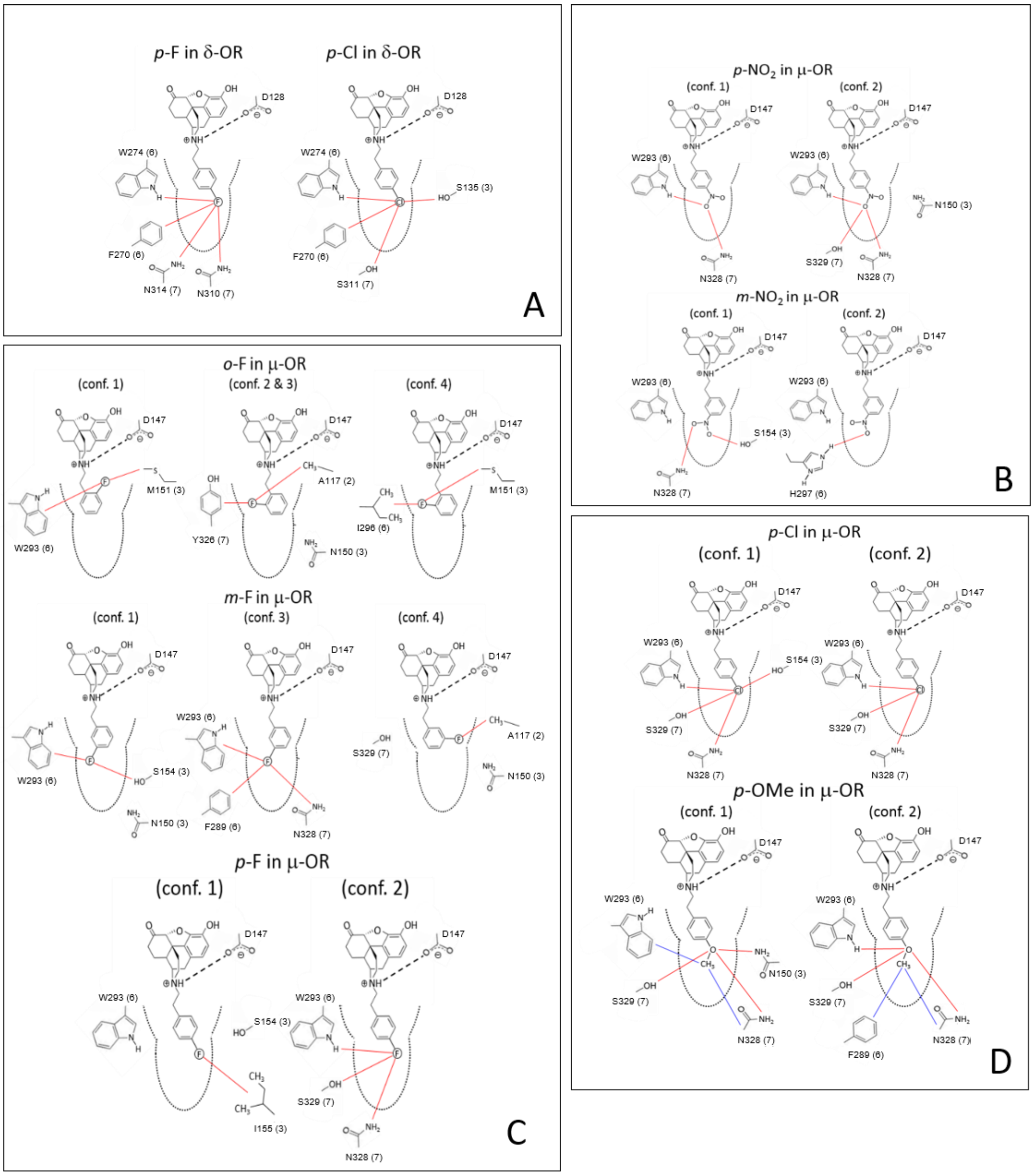

Figure S3. Critical tail-OR interactions (in red lines, statistically significant) of substitutions leading to the differences in activity and/or potency reported in Table I. (A) *p*-F (weak partial δ agonist) vs. *p*-Cl (potent full δ agonist). (B) *p*-nitro (potent partial μ agonist) vs. *m*-nitro (μ antagonist). (C) *p*-F and *m*-F (potent partial μ agonists) vs. *o*-F (potent full μ agonist). (D) *p*-F, *p*-nitro and *p*-Cl (all potent partial μ agonists) vs. the *p*-methoxy (μ antagonist). When a residue is shown near the pocket with no connecting red lines, it means its contact with the substituent group is infrequent (although not negligible) and may be strengthened with suitable modifications of the substituent. All the conformers of the ligands are stabilized by the pocket; only those with the highest stabilization (predicted based on the number and frequency of polar/H-bond/halogen-bond contacts) are shown.

Scheme S1. Synthesis of Hydromorphone (**S5**), *N*-Norhydromorphone (**S10**) and *N*-Phenethylnorhydromorphone (**S11**). Reaction Conditions and Yield (%)

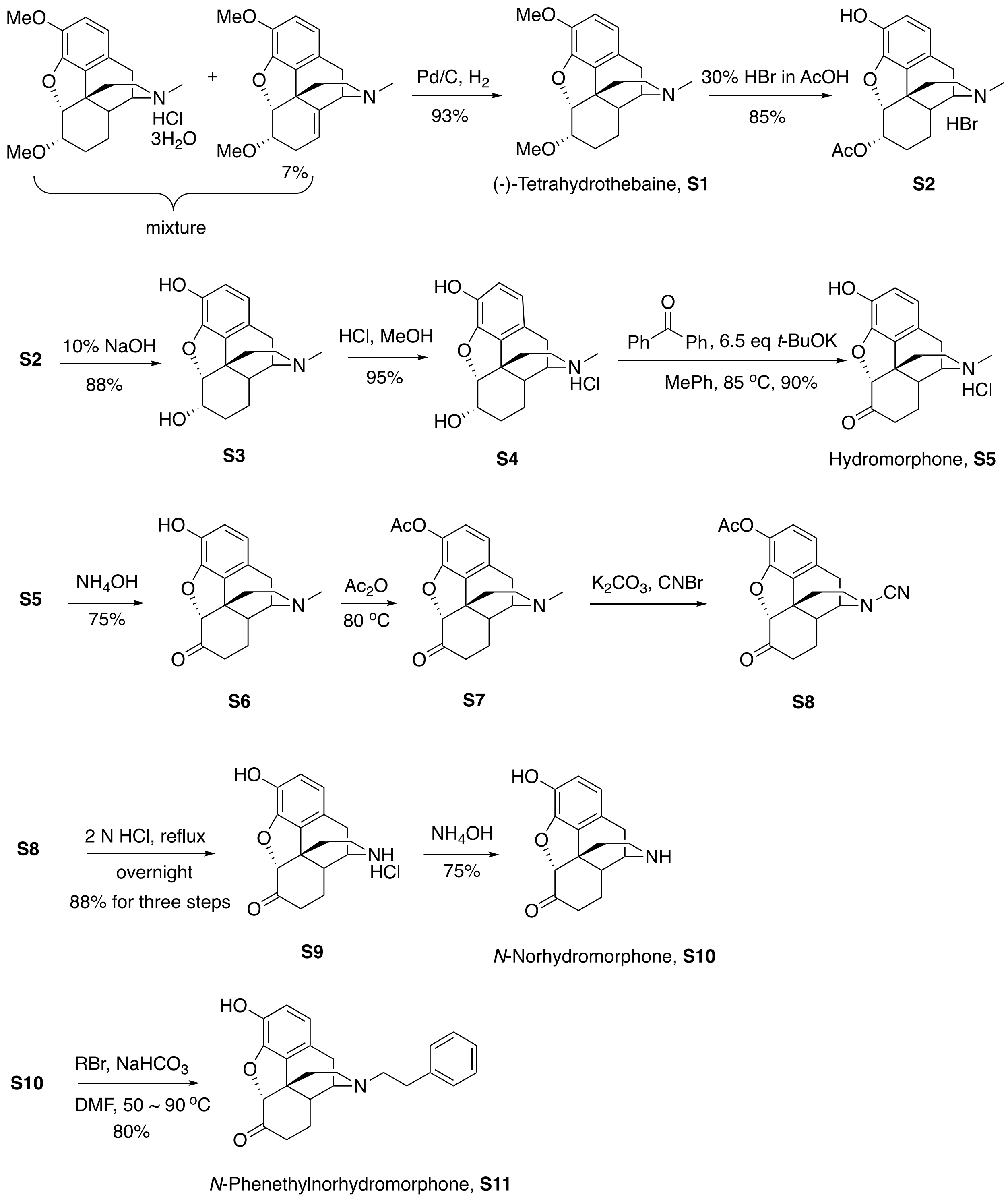

Supplement: Supplementary file 1 [file molecules-25-02640-s001.pdf]
